# Troponin destabilization impairs sarcomere-cytoskeleton interactions in iPSC-derived cardiomyocytes from dilated cardiomyopathy patients

**DOI:** 10.1038/s41598-019-56597-3

**Published:** 2020-01-14

**Authors:** Yuanyuan Dai, Asset Amenov, Nadezda Ignatyeva, Andreas Koschinski, Hang Xu, Poh Loong Soong, Malte Tiburcy, Wolfgang A. Linke, Manuela Zaccolo, Gerd Hasenfuss, Wolfram-Hubertus Zimmermann, Antje Ebert

**Affiliations:** 1 Heart Center, Department of Cardiology and Pneumology, Goettingen, Germany; 2 Institute of Pharmacology, University of Goettingen, Robert-Koch-Str. 40, 37075 Goettingen, Germany; 3 DZHK (German Center for Cardiovascular Research), partner site, Goettingen, Germany; 4 Institute of Physiology II, University of Muenster, Muenster, Germany; 5 Department of Physiology, Anatomy and Genetics, University of Oxford, Oxford, OX1 3PT UK

**Keywords:** Cytoskeleton, Mechanisms of disease, Phosphorylation

## Abstract

The sarcomeric troponin-tropomyosin complex is a critical mediator of excitation-contraction coupling, sarcomeric stability and force generation. We previously reported that induced pluripotent stem cell-derived cardiomyocytes (iPSC-CMs) from patients with a dilated cardiomyopathy (DCM) mutation, troponin T (TnT)-R173W, display sarcomere protein misalignment and impaired contractility. Yet it is not known how TnT mutation causes dysfunction of sarcomere microdomains and how these events contribute to misalignment of sarcomeric proteins in presence of DCM TnT-R173W. Using a human iPSC-CM model combined with CRISPR/Cas9-engineered isogenic controls, we uncovered that TnT-R173W destabilizes molecular interactions of troponin with tropomyosin, and limits binding of PKA to local sarcomere microdomains. This attenuates troponin phosphorylation and dysregulates local sarcomeric microdomains in DCM iPSC-CMs. Disrupted microdomain signaling impairs MYH7-mediated, AMPK-dependent sarcomere-cytoskeleton filament interactions and plasma membrane attachment. Small molecule-based activation of AMPK can restore TnT microdomain interactions, and partially recovers sarcomere protein misalignment as well as impaired contractility in DCM TnT-R173W iPSC-CMs. Our findings suggest a novel therapeutic direction targeting sarcomere- cytoskeleton interactions to induce sarcomere re-organization and contractile recovery in DCM.

## Introduction

Sarcomeres are the basic contractile unit of cardiac cells, whose particularly specialized function depends on a highly organized structure. The troponin-tropomyosin (Tn-Tm) complex at sarcomeric thin filaments is a critical component for excitation-contraction coupling. Stability and anchoring of the Tn-Tm complex on sarcomeres is provided by binding of the troponin T (TnT) subunit to Tm and the troponin I subunit (TnI) on actin myofilaments. Tropomyosin (TPM) together with TnI regulates actin/myosin binding and ATPase function in presence of micromolar, cytocolic Ca^2+^, which is bound by the troponin C subunit (TnC). This highly sensitive mechanisms is fine- tuned by post-translational modifications, such as PKA-mediated phosphorylation of TnI. Mutations in the Tn-Tm complex lead to severe disease, such as dilated cardiomyopathy (DCM). DCM is featured by left ventricular dilatation, contractile dysfunction, and arrhythmias^1^ and represents a frequent cause of heart failure. More than 25% of DCM cases are caused by inherited mutations, particularly in sarcomeric proteins^2^. Recently, human iPSC-derived cardiomyocytes (iPSC-CMs) have been utilized for human genetic disease modeling^3–6^ and drug testing^7^. Here, we analyze a sarcomeric mutation in cardiac troponin T (TnT), TnT-R173W. This mutation is located within one of the two tropomyosin binding regions of TnT, the T1 domain^8^ and was the first DCM mutation reported in a human patient-specific iPSC-derived cardiomyocyte model^9^. This report demonstrated DCM patient-specific iPSC-CMs to display molecular disease-specific phenotypes such as abnormal sarcomeric structure, dysregulated Ca^2+^ signaling, and impaired contractility^9^. More recently, disturbed beta-adrenergic signaling due to epigenetic modulation has been attributed as a source for PDE2A and PDE3A upregulation, as well as dysregulated Ca^2+^ handling^10^. These and other studies^11–14^ show that sarcomeric microdomain organization plays an important role in maintenance of cardiomyocyte structure and function. Likewise, sarcomeric microdomains are critical for cytoskeleton filament integrity and mediate attachment with the plasma membrane^15^. Previously, cytoskeletal interactions with caveolin-enriched plasma membrane domains have been implied in sarcomere attachment as well as in signal transduction during heart failure^16^. Novel proteins contributing to these processes have been recently characterized, such as AMP-activated protein kinase (AMPK)^17,18^. The regulation of metabolism by AMPK in various cell types is well recognized, as well as the ability of AMPK to sense ATP levels^18–20^. Of note, AMPK has been shown to also act as a cytoskeleton remodeling protein^21–23^. Despite this progress, the contribution of cardiomyopathy mutations, such as DCM TnT-R173W, to dysfunction of local sarcomeric microdomains and their cytoskeleton/plasma membrane interactions is not yet clear.

Here, we uncover novel mechanistic features underlying disorganization of sarcomeric protein- and microdomain array, as well as reduced contractility in DCM patient-specific iPSC- CMs. We generated CRISPR/Cas9 gene edited troponin T knock-out (TnT-KO) iPSC-CMs as an isogenic control for specificity. We report disturbed molecular interactions of troponin and tropomyosin in patient-specific iPSC-CMs carrying the DCM-TnT-R173W mutation, compared to gene edited TnT-KO iPSC-CMs and healthy controls (TnT-WT). Importantly, the DCM mutation TnT-R173W destabilizes TnT interactions with PKA, resulting in diminished troponin I (TnI) phosphorylation. This contributes to impaired force generation as well as reduced contractility, which we validated in a 3D engineered heart muscle (EHM) model. A Foerster Resonance Electron Transfer (FRET)-based molecular sensor and interrogation of sarcomeric PDE activity revealed dysregulation of local TnT microdomains in presence of DCM-TnT-R173W, which results in impaired interactions with cytoskeleton filaments as well as reduced plasma membrane attachment. We identified AMPK to assist in cytoskeleton filament interactions with both sarcomere- and plasma membrane junctions via myosin heavy chain 7 (MYH7) in DCM iPSC-CMs. We showed that AMPK activation can in part overcome destabilized microdomain interactions, as well as sarcomere protein misalignment and impaired contractility in presence of the TnT-R173W mutation. Our studies present new information regarding disturbed troponin complex interactions in patient-specific iPSC-CMs carrying an inherited DCM mutation. We contribute to novel understanding of local signal regulation in sarcomeric microdomains as well as sarcomere interactions with other cytoskeleton filament proteins and plasma membrane compartments. These findings may be exploited in the future for therapeutic manipulation of molecular disease mechanisms.

## Results

### Patient-specific iPSC-CMs and engineered heart muscle (EHM) recapitulate sarcomere protein misalignment and impaired contractility in presence of DCM-TnT-R173W

**Figure 1:**
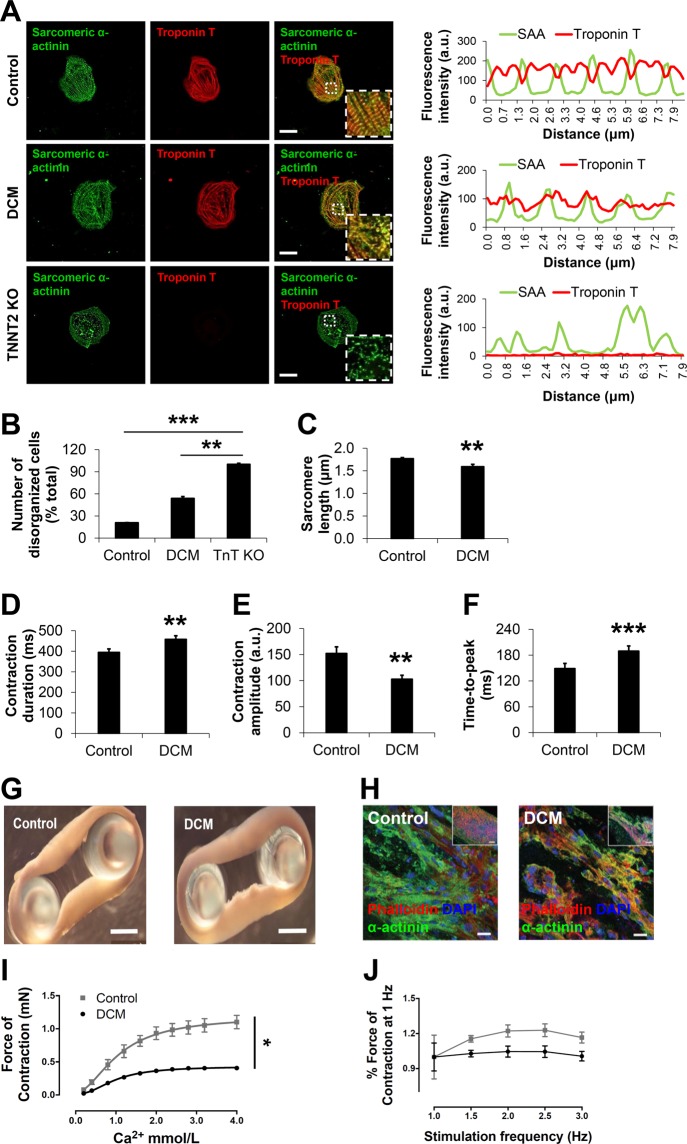
DCM iPSC-CMs display impaired sarcomeric protein alignment and reduced contractility. (**A,B**) Analysis of sarcomere protein alignment in DCM and TnT KO iPSC-CMs, compared to healthy controls. (**A**) Immunohistochemistry for sarcomeric alpha-actinin (SAA) and cardiac troponin T (TnT), followed by confocal imaging (scale bar, 20 μm). Line scans show striation patterns for SAA and TnT sarcomeric distributions (y-axis, fluorescence intensity in arbitrary units; x-axis, distance in μm). (**B**) Quantification of disorganized iPSC-CMs using confocal images as shown in Fig. 1A. Control, n = 70 cells, 3 cell lines; DCM, n = 92 cells, 3 cell lines; TnT KO, n = 36 cells, 1 cell line. **P < 0.01, ***P < 0.001 (one-way ANOVA and Sidak’s multiple comparisons test). (**C**) DCM TnT-R173W iPSC-CMs display significantly reduced sarcomere length, compared to healthy controls. Data are expressed as mean ± s.e.m. Control (WT), n = 3 cell lines and 39 images were analyzed; DCM (TnT-R173W), n = 3 cell lines and 42 images were analyzed. (**D–F**) Motion-based contractility analysis of iPSC-CMs revealed significantly prolonged contraction duration (**D**), *P* = 0.01; reduced contraction amplitude (**E**), *P* = 0.01; and prolonged time to peak (**F**), ***P < 0.001. Data are expressed as mean ± sem. Control (WT), n = 3 cell lines; DCM, n = 3 cell lines. **P < 0.01 as calculated by Student’s t-test. (**G–J**) TnT- R173W results in impaired contraction force in human iPSC-derived engineered heart muscle (EHM) from DCM iPSC-CMs and healthy controls. (**G**) Mechanical loading of control and DCM EHMs on PDMS stretchers before force testing (scale bars: 2 mm). (**H**) Immunofluorescence images of EHMs show distribution and alignment of iPSC-CMs (sarcomeric alpha-actinin) for healthy control and DCM (scale bars: 20 μm; inset: 100 μm). (**I**) EHMs were attached to force transducers and pre-stretched to reach optimal sarcomere length (maximal isometric active tension according to Frank-Starling-mechanism). DCM EHMs demonstrate reduced force of contraction (FOC) under isometric conditions and addition of increasing Ca^2+^ concentrations (*P* < 0.05). (**J**) Shown is the response to electrical stimulation for control- and DCM EHMs (*P* = ns). n = 8 EHMs per group. Data are expressed as mean ± sem. *P < 0.05 and ns, not significant as calculated by two-way ANOVA and Tukey’s post-hoc test.

We employed DCM patient-specific iPSCs from a family cohort carrying an inherited DCM mutation, TnT-R173W (Fig. 1A)^9^ as well as healthy control (WT) iPSCs. We generated an isogenic TnT knock-out iPSC line as a negative control, using site-specific CRISPR- Cas9 gene editing to induce a frameshift mutation at exon 2 (Fig. 1A, Supplementary Fig. 1A,B). Human iPSC lines displayed regular expression of pluripotency markers (Supplementary Fig. 1C). Next, TnT-KO as well as DCM TnT-R173W and WT iPSCs were differentiated to iPSC-CMs using a small molecule-based monolayer protocol described earlier^24–26^ (Fig. 1, Supplementary Fig. 1D,E).

While beating iPSC-CMs were observed for healthy control and DCM TnT-R173W patient- specific iPSC-CM lines, differentiated iPSC-CMs from TnT-KO iPSCs did not express TnT (Fig. 1A) and did not display any beating (Movies S1–3. To establish DCM-specific phenotypic features in DCM TnT-R173W iPSC-CMs compared to TnT-KO and healthy controls, we first analyzed sarcomere protein arrangement. The number of cells with severely abnormal sarcomeres was significantly increased in DCM TnT-R173W iPSC-CMs compared to healthy controls (Fig. 1A,B). No cells with organized sarcomeres were found in TnT-KO iPSC-CMs (Fig. 1A,B). Of note, DCM TnT-R173W iPSC-CMs showed significantly reduced sarcomere length (Fig. 1C) and a less negative correlation coefficient corresponding to diminished sarcomere protein regularity (Supplementary Fig. 1F), compared to healthy controls.

Contractility of DCM TnT-R173W iPSC-CMs was analyzed by an automated high-speed imaging platform, MuscleMotion^27^ (Fig. 1D–F). DCM TnT-R173W iPSC-CMs displayed significantly prolonged contraction duration (Fig. 1D), reduced amplitude (Fig. 1E) and increased time-to-peak (Fig. 1F), compared to healthy control (TnT-WT) iPSC-CMs. Contractility analysis confirmed TnT-KO iPSC-CMs to be unable to contract. To further corroborate that destabilization of TnT complex interactions in presence of TnT-R173W results in reduced contractile force, we utilized a 3D model of contracting human engineered heart muscle (Fig. 1G–J). EHMs from both DCM TnT-R173W iPSC-CMs and healthy control iPSC-CMs (TnT-WT) stained in cross-sections for cardiac marker proteins such as sarcomeric alpha-actinin (Fig. 1G,H). TnT-KO iPSC-CMs were unable to condense into EHM rings, likely due to poor stability and integrity of TnT-KO iPSC-CM sarcomeres. Therefore, TnT-KO EHMs could not be generated. The contraction force in EHMs was determined over a range of Ca^2+^ concentrations (Fig. 1I,J) EHMs from DCM TnT-R173W iPSC-CMs showed significantly reduced force of contraction, compared to WT controls (Fig. 1I), and the response to electrical stimulation was slightly lower in DCM EHMs versus WT control EHMs following pacing at 1 Hz (Fig. 1J).

### Interactions of the TnT complex are disturbed in DCM TnT-R173W iPSC-CMs

We next compared consequences of the disease-specific mutation TnT-R173W versus TnT- KO for the expression of the troponin complex subunits in iPSC-CMs at the gene and protein levels. First, we confirmed that mRNA expression of TNNT2, TNNI3, TNNC1 and TPM1 in DCM TnT-R173W iPSC-CMs was not significantly different from WT controls (Supplementary Fig. 2A–D). In contrast, TNNT2 KO iPSC-CMs were found to not express TNNT2. In line with previous publications^28^, we found TNNT2 KO to result in loss of TnI expression at both mRNA and protein levels (Supplementary Fig. 2,D–H)^28^. TnT-KO iPSC-CMs also displayed a substantial loss of TnC (Supplementary Fig. 2,B–F)^28^ and Tm (Supplementary Fig. 2C–G) at mRNA and protein levels. On the other hand, protein expression levels of TnI, TnC and Tm were not significantly altered in DCM patient-specific iPSC-CMs versus controls (Supplementary Fig. 2E–H).

**Figure 2:**
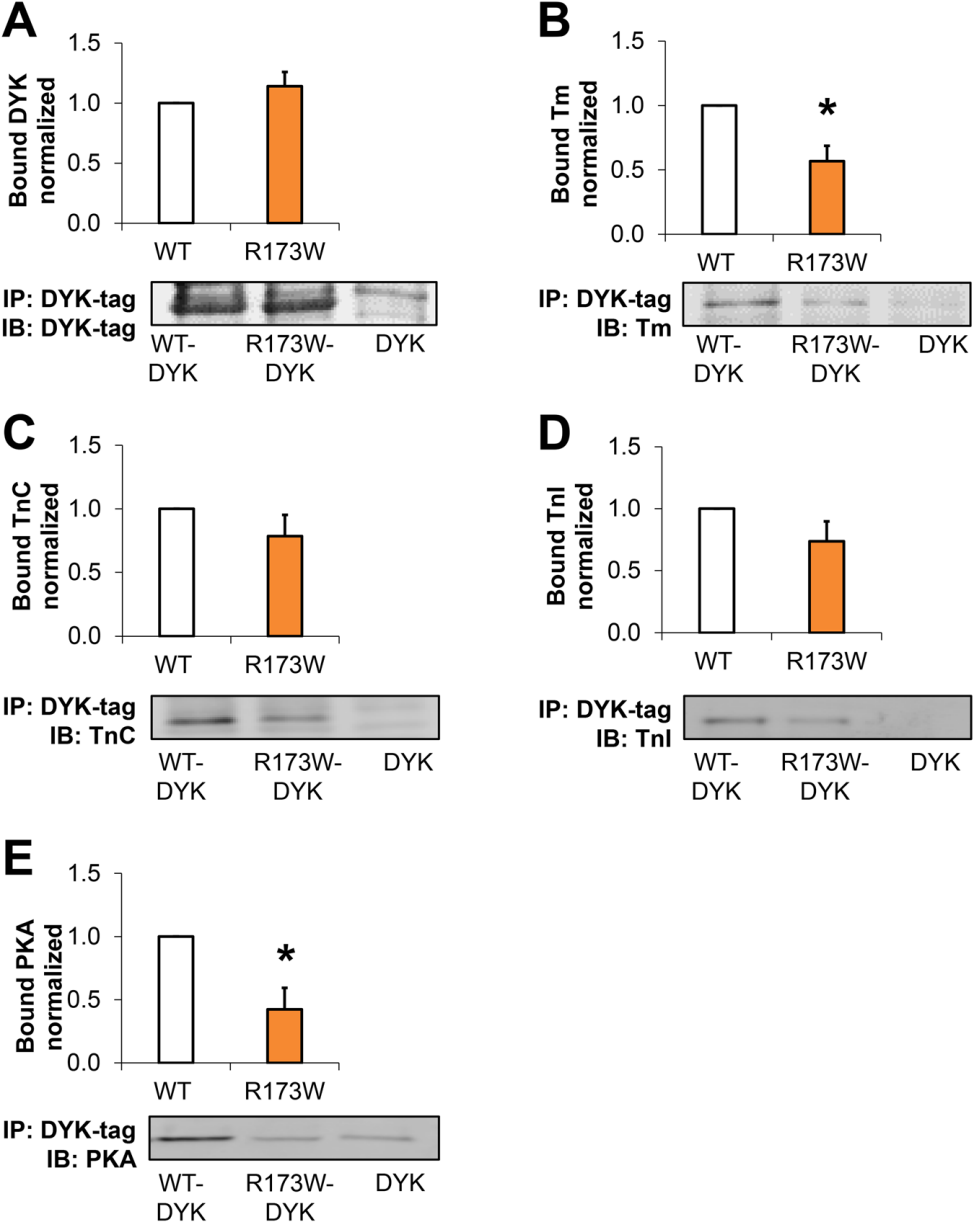
Troponin complex interactions are disturbed in presence of DCM-TnT-R173W. **(A–D)** Co-immunoprecipitation showed reduced capacity of TnT-R173W to bind to tropomyosin, compared to TnT-WT. Human TnT-R173W-DYK, TnT-WT-DYK or DYK (negative control) was overexpressed in HEK cells and immobilized on DYK-antibody-decorated beads. Human iPSC- CM lysate from healthy controls was used for co-immunoprecipitation to test the binding capacity of TnT-WT or TnT-R173W towards troponin complex subunits and tropomyosin. (**A**) Binding of TnT-WT-DYK and TnT-R173W-DYK to DYK-coated beads is comparable. TnT-R173W binding to tropomyosin (Tm) (**B**) is significantly reduced compared to TnT-WT. (**C**) Binding of TnT-DYK to TnC (**D**) Binding of TnT-DYK to TnI. (**E**) PKA binding is significantly reduced in TnT- R173W-DYK, compared to TnT-WT-DYK. Representative membrane scans are shown. Bargraphs show averages of n = 8 experiments for TnT-DYK; n = 5 experiments for Tm; n = 6 experiments for TnC; n = 5 experiments for TnI; n = 4 experiments for PKA. Groups in (**A–E**) are shown following subtraction of respective DYK negative controls. Bound protein was normalized by immobilized TnT-WT-DYK or TnT-R173W-DYK (see also Supplementary Fig. 4A,B). Data are expressed as mean ± sem. *P < 0.05 (one sample t- and Wilcoxon test).

We next probed if the troponin complex subunits and tropomyosin would interact in presence of the TnT-R173W mutation and following TnT-KO in the same manner as in healthy control iPSC-CMs (TnT-WT). Co-immunoprecipitation experiments indicated reduced binding capacity of TnT-R173W towards Tm, compared to iPSC-CMs (Supplementary Fig. 3A–C). TnT-KO iPSC-CMs were utilized as a control for binding specificity (Supplementary Fig. 3,A–D). To further corroborate these findings, we employed *in-vitro* interaction studies with recombinant flag (DYK)-tagged TnT-WT and TnT-R173W. TnT-WT-DYK, TnT-R173W-DYK or DYK as a negative control were expressed in HEK 293 T cells, immobilized on flag-decorated beads (Fig. 2A) and incubated with iPSC-CM lysate from healthy controls (Supplementary Fig. 4A). Binding of TnC, TnI and Tm was determined in bound fractions via immunoblot (Fig. 2B–D, Supplementary Fig. 4A). A flag-tag encoding vector was utilized as a negative control for overexpression- and binding studies (Fig. 2A–E, Supplementary Fig. 4A). Of note, TnT- binding to Tm, which anchors the troponin complex on the actin myofilaments, was significantly reduced in presence of the TnT-R173W mutation, compared to TnT-WT (Fig. 2B). Particularly, tropomyosin binding is critical for force transduction by gate-keeping the myosin-binding site on actin, as well as sarcomere stability^29^. Our findings suggest that the DCM TnT-R173W mutation destabilizes the troponin-tropomyosin interaction.

### Dysregulated sarcomeric PKA function and elevated sarcomeric cAMP are consequences of the DCM mutation TnT-R173W

The troponin complex is a key regulator of calcium binding and contractility in cardiomyocytes, to which PKA-mediated phosphorylation contributes. TnT acts as an A-kinase anchoring protein (AKAP)^30^. We thus investigated if the DCM TnT-R173W mutation impairs PKA binding on sarcomeric myofilaments. Immunoprecipitation studies showed that TnT-R173W binds significantly less PKA than WT-TnT (Fig. 2E, Supplementary Fig. 4A). Moreover, these data are in line with a previous study reporting lower PKA activity in DCM TnT-R173W iPSC-CMs^10^.

**Figure 3:**
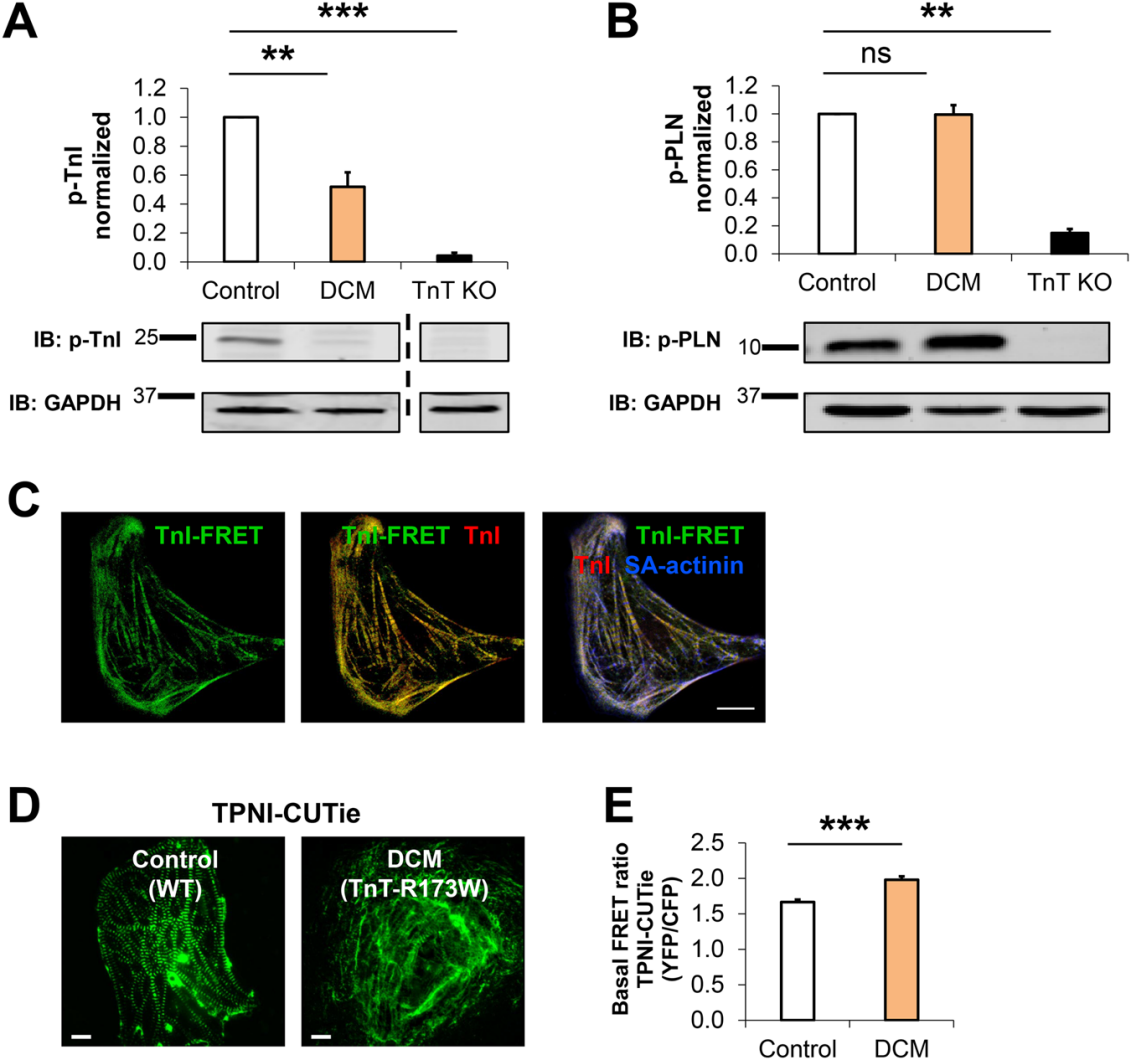
Reduced PKA-mediated TnI phosphorylation in TnT-R173W iPSC-CMs cannot be compensated by local sarcomeric cAMP modulation. (**A**) PKA-mediated phosphorylation of TnI at Ser 23/24 is reduced in DCM iPSC-CMs, as well as TnT KO iPSC-CMs. Bargraph presents averages of n = 5 experiments for control (WT, n = 3 cell lines), DCM (TnT-R173W, n = 3 cell lines), and TnT KO (n = 1 cell line); shown below are representative immunoblots. Data are expressed as mean ± sem. **P < 0.01, ***P < 0.001 and ns, not significant (one sample t- and Wilcoxon test). (**B**) Phosphorylation of PLN is not altered in DCM iPSC-CMs compared to healthy controls. Bar graph presents averages of n = 6 experiments for control (WT, n = 3 cell lines), DCM (TnT-R173W, n = 3 cell lines), and TnT KO (n = 1 cell line); representative immunoblots are shown below. Data are expressed as mean ± sem. **P < 0.01 and ns, not significant (one sample t- and Wilcoxon test). (**C–E**) TPNI-FRET-sensor-based detection of cAMP levels in TnT-WT and TnT-R173W iPSC-CMs. Shown are basal FRET ratios (emission YFP/emission CFP following background subtraction). (**C**) Sarcomeric localization of the TPNI-CUTie FRET-sensor in iPSC-CMs. TPNI-FRET signal co-localizes with TnI co-staining in immunohistochemistry. Co-staining for sarcomeric alpha-actinin is shown. Scale bar, 10 μm. (**D**,**E**) Detection of local cAMP upregulation at the Tn complex via the TPNI-FRET-sensor in DCM-TnT- R173W iPSC-CMs, compared to control iPSC-CMs (WT). (**D**) Representative images are shown; scale bar, 10 μm. (**E**) Quantification of (**D**). WT iPSC-CMs (healthy control), n = 45 cells; TnT-R173 iPSC-CMs (DCM), n = 45 cells. ***P < 0.001 (Student’s t- test). Data are shown as mean ± sem.

These findings pointed to a local modulation of sarcomeric functions at the TnT complex in presence of the DCM mutation TnT-R173W. As PKA directly interacts with the troponin complex and phosphorylates TnI, we considered that altered PKA levels at the sarcomere would affect phosphorylation levels of TnI, which in turn contributes to regulating contractility^31^. We therefore tested phosphorylation of TnI-Ser 23/24 in DCM patient-specific TnT-R173W iPSC- CMs, compared to healthy control iPSC-CMs and TnT-KO iPSC-CMs. In DCM iPSC-CMs, substantially reduced phosphorylation of TnI was detected (Fig. 3A, Supplementary Fig. 4B). In TnT-KO iPSC-CMs, low phosphorylation of TnI-Ser 23/24 was observed (Fig. 3A), in line with very low baseline expression of TnI in TnT-KO iPSC-CMs (Supplementary Fig. 2H). Of note, reduced sarcomeric target phosphorylation was not a ubiquitous effect. PKA-dependent phosphorylation of phospholamban (Pln) was not significantly altered in presence of TnT-R173W in DCM patient-specific iPSC-CMs, compared to WT iPSC-CMs (Fig. 3B).

To further explore local regulation of TnT complex function in relation to PKA, we next tested if DCM-TnT-R173W leads to alterations in local cAMP levels at the sarcomere in comparison to cytosolic cAMP. We employed a FRET (Foerster Resonance Electron Transfer)-based readout for detection of locus-specific cAMP levels at the TnT complex. A targeted cAMP FRET-biosensor, CUTie, which has the PKA cyclic nucleoside binding domain fused to TnI (TnI-CUTie)^14^ was utilized for measurement of cAMP at sarcomeric myofilaments. The TnI-CUTie sensor recapitulated correctly targeted TnI-CUTie biosensor in patient-specific and healthy control iPSC- CMs (Fig. 3C–E) while in contrast, severely disorganized sarcomeric structure was detected in TnT-KO iPSC-CMs (Supplementary Fig. 4C,D). Investigation of local sarcomeric cAMP levels in DCM TnT-R173W iPSC-CMs versus healthy controls revealed a slight increase of sarcomeric cAMP (Fig. 3D,E). This may present a local compensatory reaction in DCM iPSC-CMs to increase PKA activity and TnI phosphorylation. However, given diminished PKA anchoring and resulting reduced phosphorylation of TnI in presence of TnT-R173W, slightly elevated sarcomeric cAMP is not sufficient to activate the remaining AKAP-bound sarcomeric PKA. These findings highlight local microdomain regulation of PKA function at the sarcomere in presence of the DCM TnT-R173W mutation.

### Local dysfunction at the TnT complex in presence of TnT-R173W results in disrupted sarcomere-cytoskeleton filament- and plasma membrane interactions

**Figure 4:**
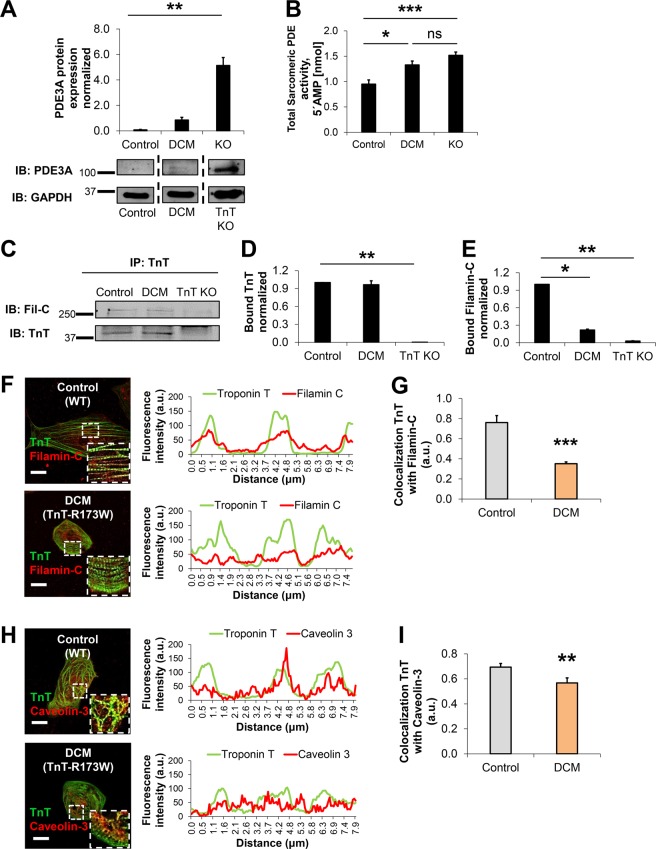
Sarcomeric microdomain regulation and interactions with cytoskeleton filament proteins are disturbed in DCM-TnT-R173W iPSC-CMs. (**A**) PDE3A protein expression levels are increased in DCM iPSC-CMs compared to healthy controls. (**B**) Total sarcomeric PDE activity is significantly increased in DCM- and TnT KO iPSC-CMs, compared to healthy controls. (**A,B**) Averages of 3 experiments are shown for control (n = 2 cell lines), DCM (n = 2 cell lines) and TnT- KO (n = 1 cell line). Representative membrane scans are shown. Data are shown as mean ± sem. *P < 0.05, ***P < 0.01, **P < 0.001, ns = not significant as calculated by Kruskal-Wallis test and Dunn’s multiple comparisons test. (**C–E**) Interactions of sarcomeric TnT with the z-disc and cytoskeleton protein, filamin-C, were assessed by immunoprecipitation using a TnT-specific antibody. (**C**) Representative membrane scans for immunoprecipitation of filamin-C with TnT are shown. (**D**) Equal amounts of TnT-WT and TnT- R173W were immunoprecipitated from lysates of healthy control and DCM iPSC-CMs. (**E**) Binding of filamin-C to DCM-TnT-R173W is reduced, compared to healthy control WT-TnT. Bargraphs shown averages of n = 2 experiments for control (WT, n = 2 cell lines), DCM (TnT- R173W, n = 3 cell lines), and TnT KO (n = 1 cell line). Bound TnT was normalized by input and GAPDH (see also Supplementary Fig. 6A). *P < 0.05, **P < 0.01, ***P < 0.001 and ns, not significant (one sample t- and Wilcoxon test). Data are expressed as mean ± sem. (**F–I**) Co-localization of TnT with the z-disc and cytoskeleton attachment protein, filamin-C, and caveolin-3 is reduced in DCM iPSC-CMs compared to healthy controls. Immunostainings, confocal images and corresponding ImageJ-based quantifications are shown. Line scans show striation patterns for sarcomeric distribution of TnT (y-axis, fluorescence intensity in arbitrary units; x-axis, distance in μm). (**F**) Co-localization of filamin-C with TnT and (**G**) Quantification of (**F**). Analysis was done for WT healthy control, n = 1 cell line and n = 17 cells, 16 images, as well as DCM, n = 1 cell line and n = 48 cells, 25 images. (**H**) Co-localization of TnT with caveolin-3 and (**I**) Quantification of (**H**). Analysis was done for WT healthy control, n = 2 cell lines and n = 38 cells, 31 images, and DCM, n = 2 cell lines and n = 44 cells, 35 images. Data are shown as mean ± sem. **P < 0.01, ***P < 0.001, as calculated by Student’s t-test.

In line with a previous report^10^ we found increased phosphodiesterase PDE3A in TnT-R173W iPSC-CMs, compared to healthy controls (Fig. 4A). Adenylyl cyclase (AC) expression levels were not significantly altered between DCM iPSC-CMs and healthy controls (Supplementary Fig. 5A). We speculated that upregulation of sarcomeric cAMP in DCM TnT-R173W iPSC-CMs may be mediated by local regulation at sarcomeric microdomains due to disrupted sarcomere protein alignment. To test this hypothesis, we first assessed sarcomeric PDE enzymatic activity by measuring 5′AMP released in DCM TnT-R173W iPSC-CMs as well as healthy controls and TnT-KO (Fig. 4B, Supplementary Fig. 5B). Overall sarcomeric (Fig. 4B) PDE activity was significantly increased in DCM TnT-R173W iPSC-CMs, compared to healthy controls (Figure 4B), while cytosolic PDE activity was not found to be significantly altered (Supplementary Figure. 6C). These experiments reveal that beta-adrenergic responsiveness in DCM iPSC-CMs is not only limited by generic cellular cAMP levels. Importantly, DCM TnT-R173W results in reduced binding of PKA to TnT-R173W as well as lower TnI phosphorylation^11^ and causes impaired sarcomeric microdomain function in DCM iPSC-CMs. This cannot be overcome by a compensatory minor increase of local sarcomeric cAMP (Fig. 3C,D). Moreover, these findings are in line with a previous report confirming that elevated PDE2A/3A caused impaired beta- adrenergic signaling in DCM iPSC-CMs^10^. We speculated that altered sarcomeric microdomain function in DCM TnT-R173W iPSC-CMs could affect sarcomere protein alignment and interactions with other cytoskeleton filament proteins as well as the plasma membrane (PM). We therefore assessed interactions of sarcomere microdomains with cytoskeleton filament proteins and the PM in more detail. Of note, we found impaired integrity of sarcomere-cytoskeleton filament junctions in DCM TnT-R173W iPSC-CMs versus healthy controls (Fig. 4C–I). Immunoprecipitation studies revealed an interaction of the cytoskeleton attachment protein, filamin-C, with TnT in healthy control (WT) iPSC-CMs (Fig. 4C–E, Supplementary Fig. 6A–C). Filamin-C localizes to the sarcomeric z-disc but also to other subcellular sites, such as cytoskeleton filament microdomains and is suggested to be involved in cytoskeleton signaling and remodeling^16^. The dynamic distribution of filamin-C suggests it is exposed to TnT at z-disc interaction zones with TnT-decorated actin filaments. Interestingly, the interaction of filamin-C with TnT- R173W was significantly reduced in DCM iPSC-CMs, compared to TnT-WT controls (Fig. 4C–E, Supplementary Fig. 6A–C). We also confirmed reduced co-localization of TnT and filamin-C in DCM TnT-R173W iPSC-CMs, compared to TnT-WT controls (Fig. 4F,G). No significant difference in filamin-C expression was observed in DCM iPSC-CMs compared to healthy controls and TnT KO-iPSC-CMs (Supplementary Fig. 6B,C).

Together, these data indicate in presence of DCM TnT-R173W impaired interactions of cytoskeleton filaments with TnT-enriched sarcomere microdomains. These junctions also contribute to attachment of sarcomere- and other cytoskeleton elements to the plasma membrane, which contains caveolin-3-enriched microdomains^16^. Co-localization of caveolin-3 with filamin-C at plasma membrane microdomains has been previously reported^16^. Therefore, we compared co- localization of TnT with caveolin-3 (Fig. 4H,I) in DCM TnT-R173W iPSC-CMs versus TnT- WT controls. Interestingly, TnT-caveolin-3 co-localization was significantly reduced in DCM iPSC-CM compared to healthy controls (Fig. 4H,I). Overall, these data suggest reduced attachment of sarcomere microdomains with cytoskeleton filaments as well as the plasma membrane in DCM TnT-R173W iPSC-CMs. Disturbed sarcomere microdomain attachment is likely a consequence of the impaired interaction of TnT with tropomyosin in presence of TnT- R173W (Fig. 2A,B,E) and the resulting disrupted sarcomere protein alignment (Fig. 1A–C, Supplementary Fig. 1F).

### AMPK activation improves sarcomere-cytoskeleton attachment as well as sarcomere protein alignment

**Figure 5:**
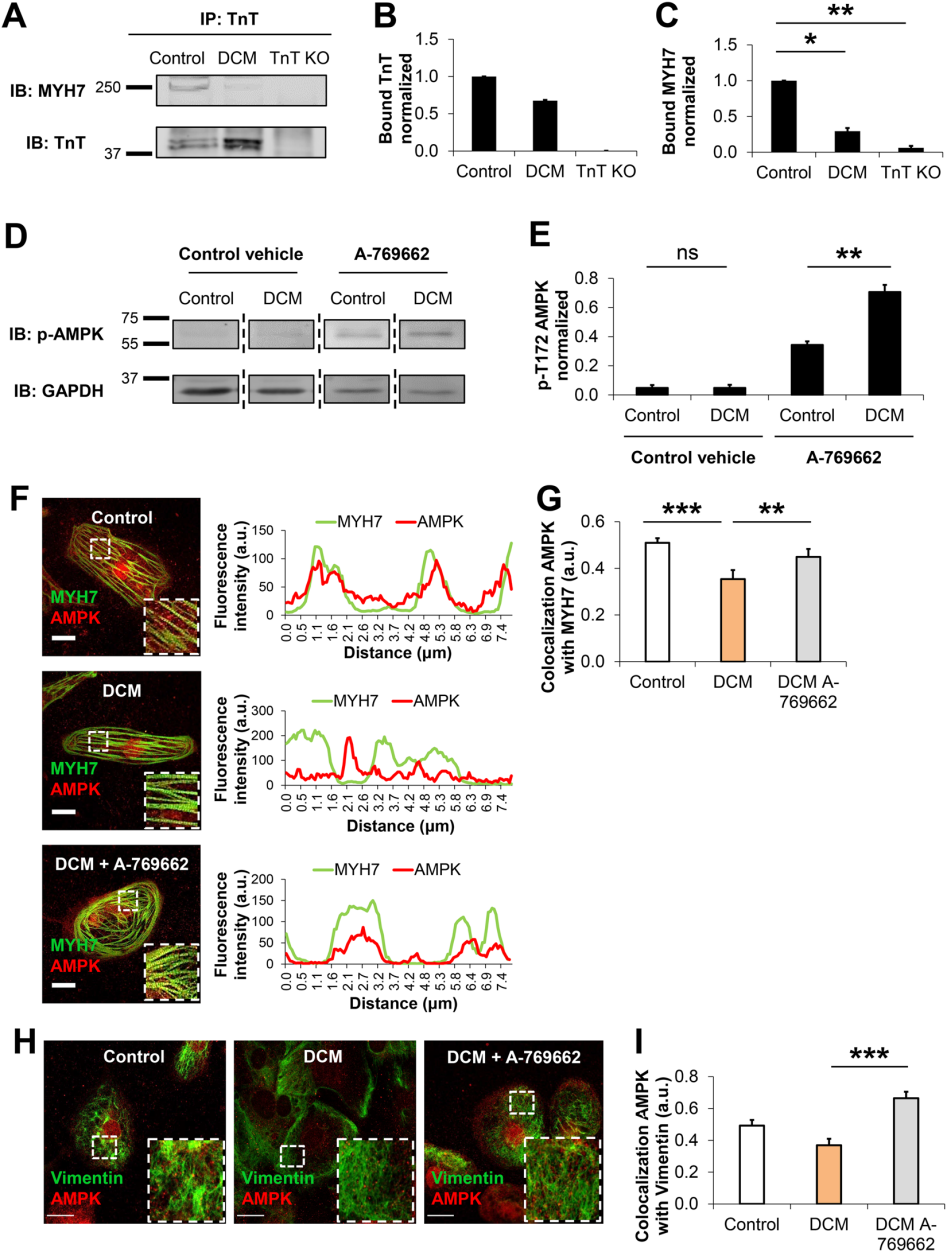
AMPK is a positive regulator of sarcomere-cytoskeleton filament interactions. (**A**–**C**) Immunoprecipitation of MYH7 with TnT is shown, using cell lysates from DCM iPSC-CMs, healthy controls and TnT KO. Interaction of TnT with MYH7 was assessed by immunoprecipitation using a TnT-specific antibody. (**A**) Representative membrane scans for immunoprecipitation of TnT and MYH7 are shown. (**B**) Comparable amounts of TnT-WT and TnT- R173W were immunoprecipitated from lysates of healthy control and DCM iPSC-CMs. (**C**) Binding of MYH7 to DCM-TnT-R173W is reduced, compared to healthy controls. Bargraphs show signal from bound IP fractions following normalization for input in each group for input and loading control. Shown are averages of 2 experiments for control (WT, n = 3 cell lines), DCM (TnT-R173W, n = 3 cell lines) and TnT KO (n = 1 cell line); input and loading control are shown in Supplementary Fig. 7A. *P < 0.05, **P < 0.01 (one sample t- and Wilcoxon test). Data are expressed as mean ± sem. **(D–E)** AMPK activity in DCM versus healthy control iPSC-CMs measured via a phospho-AMPKα (Thr172)-specific antibody. Following treatment with A-769662, AMPK activity in DCM iPSC-CMs is significantly increased, compared to control vehicle (DMSO). (**D**) Representative membrane scans are shown. (**E**) Quantification of (**D**). Shown are averages of 4 experiments for control (WT, n = 3 cell lines), DCM (TnT-R173W, n = 3 cell lines) and TnT KO (n = 1 cell line); **P < 0.01 and ns, not significant (one-way ANOVA and Sidak’s multiple comparison test) data are expressed as mean ± sem. (**F–I**) Co-localization of AMPK with MYH7 and vimentin is reduced in DCM iPSC-CMs compared to healthy controls and is recovered following AMPK activation via A- 769662. Immunostaining and confocal images (scale bar, 20 μm) as well as corresponding ImageJ-based quantifications are shown. Line scans show striation patterns for sarcomeric distribution of TnT (y-axis, fluorescence intensity in arbitrary units; x-axis, distance in μm). (**F**) Co-localization of AMPK with MYH7 and (**G**) Quantification of (**F**), ***P < 0.001 for WT control vehicle vs. DCM control vehicle, **P < 0.01 for DCM control vehicle vs. DCM-A-769662 and WT control vehicle vs. DCM-A-769662, P = not significant (one-way ANOVA and Sidak multiple comparison test). Analysis was performed for WT healthy control, n = 2 cell lines and n = 68 cells; DCM, n = 1 cell line and n = 49 cells; and DCM A-769662, n = 2 cell lines and n = 74 cells. (**H**) Co-localization of AMPK with vimentin. Scale bar, 20 μm. (**I**) Quantification of (**H**), ***P < 0.001 for DCM control vehicle versus DCM-A-769662 (one-way ANOVA and Sidak multiple comparison test) WT healthy control, n = 2 cell lines and n = 38 cells; DCM, n = 1 cell line and n = 23 cells; and DCM A-769662, n = 1 cell line and n = 14 cells. Data are shown as mean ± sem.

To further investigate the impaired sarcomere-cytoskeleton filament microdomain interactions in presence of the TnT-R173W mutation, we assessed molecular factors known to regulate sarcomere interactions with cytoskeleton filament proteins. AMP-activated protein kinase (AMPK) is an established modulator of cytoskeleton filament interactions, which is known to regulate cardiomyocyte metabolism^23,32^. AMPK-mediated metabolic regulation has been recently studied also in iPSC-CMs^33^. Of note, AMPK has been shown to act as a cytoskeleton remodeling factor mediating cytoskeleton rearrangement and polarity, via interaction with myosin heavy chain proteins (MYH7–9)^17,23,32^. As myosin heavy chain 7 (MYH7) could also interact with troponin^34^, we speculated that the presence of the DCM mutation TnT-R173W may impair TnT interaction with MYH7. Destabilization of the TnT-MYH7 interaction could in turn affect AMPK-mediated regulation of cytoskeleton filaments. To probe integrity of MYH7 binding to TnT in DCM and control iPSC-CMs, we used immunoprecipitation from iPSC-CM cell lysates (Fig. 5A–C, Supplementary Fig. 7A). In presence of DCM TnT-R173W, MYH7 binding to TnT was significantly reduced, compared to WT controls (Fig. 5A–C, Supplementary Fig. 7A), confirming that the DCM TnT-R173W mutation causes disruption of sarcomere-cytoskeleton filament interactions. We next sought to test if this would affect AMPK-mediated regulation of cytoskeleton stability and integrity. To explore if sarcomere-cytoskeleton attachment would respond to AMPK modulation, we cultured DCM iPSC-CMs in presence of a small-molecule AMPK activator, A-769662^35^, or control vehicle (Fig. 5D,E). Interestingly, while AMPK activation via A-769662 increased AMPK activity in both healthy control and DCM iPSC-CMs, the increase of AMPK activity was substantially higher in DCM iPSC-CMs than in healthy controls (DCM control vehicle vs. DCM A-769662, 14.6-fold and healthy control A-769662 vs. DCM A-769662, 2.1-fold, Fig. 5D,E). AMPK protein expression was comparable in DCM TnT- R173W iPSC-CMs and healthy controls (Supplementary Fig. 7B).

**Figure 6:**
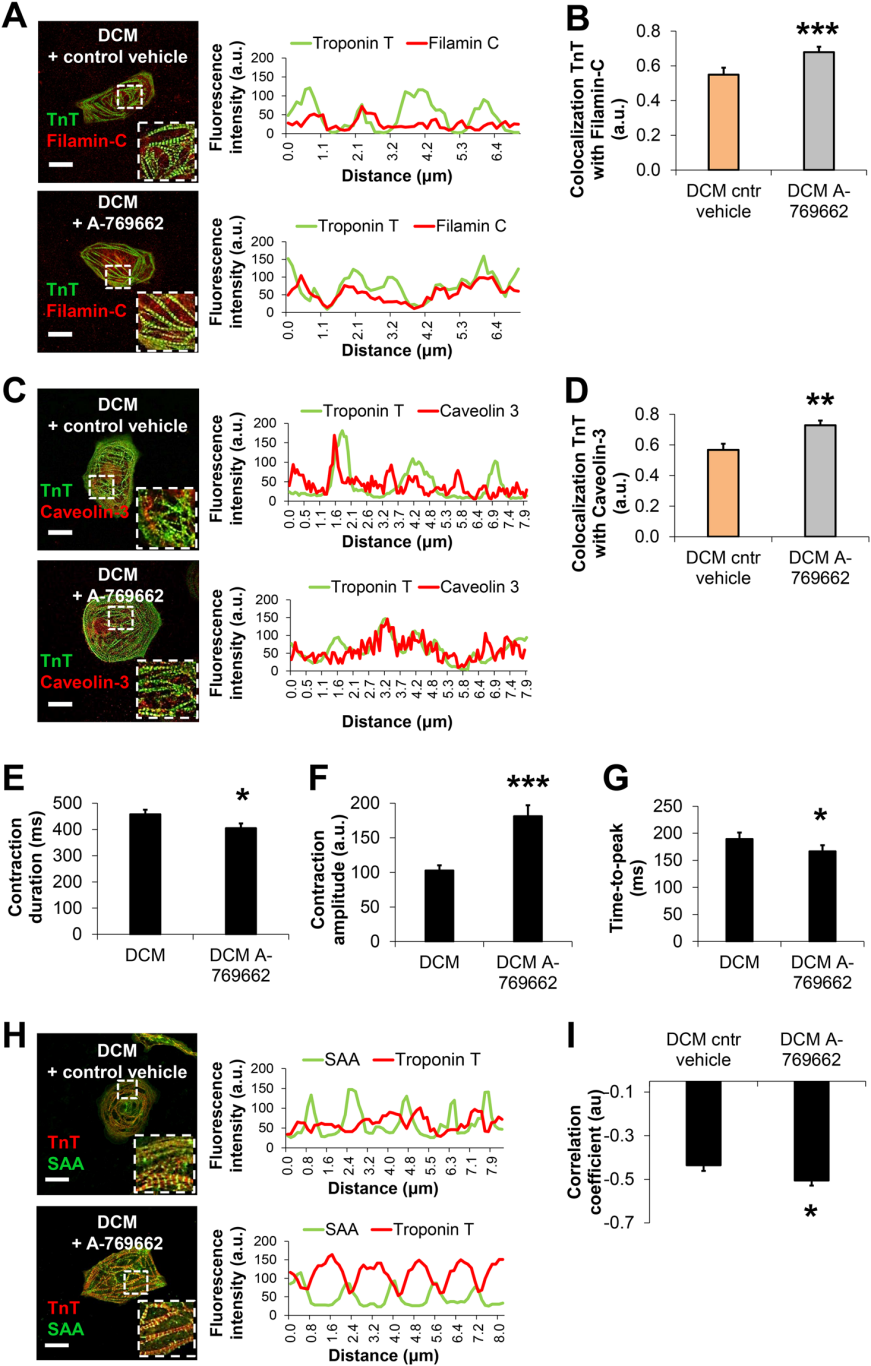
Activation of AMPK improves cytoskeleton attachment as well as sarcomere length in DCM TnT-R173W iPSC-CMs. **(A–D)** Immunostaining and confocal imaging are shown for sarcomere-cytoskeleton filament proteins in DCM iPSC-CMs following treatment with A-769662 (3 uM) or control vehicle (DMSO). Line scans show striation patterns for sarcomeric distribution of TnT (y-axis, fluorescence intensity in arbitrary units; x-axis, distance in μm). (**A**) Shown is co-localization of TnT and filamin-C. (**B**) Quantification of (**A**). Analysis was performed for DCM control vehicle, n = 2 cell lines and n = 75 cells; and DCM A-769662, n = 2 cell lines and n = 35 cells. ***P < 0.001 as calculated by Student’s t-test. Data are shown as mean ± sem. (**C**) Co- localization of TnT and caveolin-3. Scale bar, 20 μm. (**D**) Quantification of (**C**). Analysis was performed for DCM control vehicle, n = 2 cell lines and n = 44 cells; and DCM A-769662, n = 2 cell lines and n = 23 cells. Data are shown as mean ± sem. **P < 0.01, as calculated by Student’s t-test. (**E–G**) Motion-based contractility analysis of iPSC-CMs revealed following A-769662 treatment in DCM iPSC-CMs significantly reduced contraction duration (**E**), *P* = 0.01; increased contraction amplitude (**F**); ***P < 0.001; and decreased time to peak (**G**), *P* = 0.01. Data are expressed as mean ± sem. Control (WT), n = 3 cell lines; DCM, n = 3 cell lines. *P < 0.05, and ***P < 0.001 as calculated by Student’s t-test. (**H,I**) Analysis of sarcomere protein regularity is shown for confocal images of immunohistochemistry performed for DCM iPSC-CMs following 7 days of culture in presence of A-769662 or control vehicle. Line scans show striation patterns for sarcomeric distribution of TnT and SAA (y-axis, fluorescence intensity in arbitrary units; x-axis, distance in μm). (**H**) Representative confocal images are shown. Scale bar, 20 μm. (**I**) Analysis of TnT and SAA signals via Fast Fourier transformation show following A-769662 treatment improved regularity of sarcomere protein arrangement (**H**) in DCM iPSC-CMs. Analyzed were n = 31 cells for control vehicle and n = 31 cells for A-769662, n = 2 cell lines per group. Data are shown as mean ± sem. *P < 0.05 as calculated by Student’s t-test.

We next probed if increased AMPK activation in DCM iPSC-CMs could contribute to sarcomere microdomain organization as well as cytoskeleton-plasma membrane attachment in human iPSC-CMs. Interestingly, quantitative immunohistochemistry studies showed significantly reduced co-localization of MYH7 and AMPK in DCM iPSC-CMs compared to healthy controls (Fig. 5F,G). Following activation of AMPK via A-769662 in DCM iPSC-CMs, MYH7-AMPK co-localization was recovered (Fig. 5F,G). In addition, co-localization of the cytoskeleton intermediate filament protein vimentin with AMPK was significantly reduced in DCM TnT-R173W iPSC-CMs versus controls (Fig. 5H,I) and was restored by small molecule-based AMPK activation in DCM iPSC-CMs (Fig. 5H,I). These findings are in line with the diminished interaction of TnT-R173W with MYH7 (Fig. 5A–C) in DCM iPSC-CMs. We thus speculated that in DCM iPSC-CMs, the substantial increase in AMPK activity following activation via A-769662 may convene recovery of reduced sarcomere-cytoskeleton attachment. We found that following A-769662 treatment, co-localization of TnT with filamin-C (Fig. 6A,B) and caveolin-3 (Fig. 6C,D) in DCM iPSC-CMs was significantly increased, suggesting that AMPK activation supports recovery of both sarcomere-cytoskeleton- and sarcomere-PM attachment.

We next tested if AMPK activation could also recover the impaired contractility in DCM iPSC-CMs (Fig. 1D–F). Following small molecule-based activation of AMPK, we observed significantly improved contraction duration as well as amplitude and time-to-peak (Fig. 6E–G). In addition, AMPK activation recovered disrupted sarcomere protein alignment in DCM iPSC- CMs (Fig. 6H,I). Conversely, AMPK inhibition via BML-275^36^ in WT iPSC-CMs (Supplementary Fig. 8A–C) phenocopied contractile parameters observed in DCM iPSC-CMs (Fig. 1D–F), such as significantly reduced contraction amplitude, increased time-to-peak and prolonged contraction duration.

**Figure 7:**
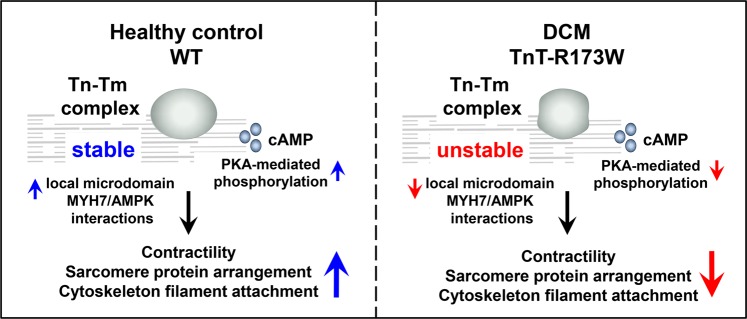
Schematic model showing consequences of impaired TnT interactions at sarcomere microdomains in presence of the DCM mutation TnT-R173W. In DCM iPSC-CMs, reduced TnT-R173W binding to tropomyosin and PKA destabilizes sarcomere protein alignment, limits TnI phosphorylation and impairs local microdomain signaling. This disrupts sarcomere-cytoskeleton filament interactions via MYH7 and AMPK, contributing to sarcomere misalignment and lowered contractility. These molecular disease phenotypes can be partially overcome by activation of AMPK.

Together, our data suggest that in DCM iPSC-CMs with a sarcomeric mutation, TnT-R173W, loss of sarcomere organization due to reduced Tm and PKA binding contributes to disrupted cytoskeleton- and plasma membrane interactions in iPSC-CMs (Fig. 7). We identified AMPK as a cytoskeleton organization protein which regulates stability of cytoskeleton filament-sarcomere interactions via MYH7. Activation of AMPK can ameliorate disrupted sarcomeric microdomain interactions in DCM patient-derived iPSC-CMs. Importantly, this recovers also DCM disease phenotypes such as reduced sarcomere protein alignment and impaired contractility in presence of a DCM mutation, TnT-R173W.

## Discussion

The molecular consequences of inherited mutations causing familial dilated cardiomyopathy (DCM) have been studied previously^2,37^. Human iPSC-CMs have been utilized for disease modeling and have revealed cellular phenotypic features of DCM in patient-derived models^9,38^. Specifically, these phenotypes comprise disrupted sarcomeric structure and abnormal calcium handling, as published earlier in a human iPSC-CM model for the DCM mutation TnT-R173W^9,10^. Moreover, impaired beta-adrenergic signaling has been characterized as an important molecular mechanism in cardiomyopathy^10,39,40^. A previous study reconstituted myofilaments containing TnT-WT or TnT-R173W and found depressed ATPase rates in presence of TnT-R173W^41^. Moreover, TnT-R173W iPSC-CMs treated with the sarcomere activator omecamtiv mecarbil^42^ showed recovered sarcomere shortening^41^. Despite this progress, many features of the molecular basis underlying these phenotypes at the sarcomere protein level are not fully understood.

Here, we utilized human patient-specific iPSC-CMs to characterize sarcomere microdomain interactions in presence of the DCM TnT-R173W mutation. We discovered binding of TnT- R173W to Tm to be reduced, which limits troponin anchoring on sarcomere filaments and destabilizes sarcomere protein alignment. A previous study which used recombinant pyrene-labeled tropomyosin did not detect any differences in the binding affinity of recombinant TnT-R173W compared to WT^43^. To our best knowledge, this is the first time a cell-based assay with iPSC-CMs is used to examine TnT binding in presence of the TnT-R173W mutation. We found in this experimental system significantly reduced binding of TnT-R173W to tropomyosin compared to WT. In addition, we found lower PKA binding at sarcomeric microdomains in presence of TnT-R173W, resulting in diminished TnI phosphorylation. Moreover, presence of the TnT-R173W mutation disturbed sarcomere microdomain-cytoskeleton filament interactions via MYH7 and AMPK, contributing to disrupted sarcomere protein alignment and impaired contractility.

Firstly, biochemical assessment revealed reduced binding of mutated TnT-R173W with Tm, which stabilizes the troponin complex on sarcomeric myofilaments. This may directly lead to the disrupted sarcomere protein alignment and regularity observed in DCM iPSC-CMs, compared to healthy controls. Moreover, reduced force generation may be an important consequence of limited TnT-Tm interaction since Tm occupies the myosin-binding site on actin filaments under low intracellular [Ca^2+^]. Reduced interaction with TnT in presence of DCM-TnT-R173W may affect correct relocation of Tm following Ca^2+^ binding to TnC as well as complete freeing of myosin- binding sites on actin, thereby limiting the initiation of contraction. We confirmed impaired force generation and contractility in presence of the DCM mutation using automated high-speed imaging-based analysis of contractility as well as a 3D engineered heart muscle (EHM) model.

Secondly, we discovered that the DCM mutation TnT-R173W contributes to dysregulation of local sarcomeric microdomains by limiting PKA binding and resulting in decreased PKA-mediated TnI phosphorylation at Ser-23/24. PKA-mediated phosphorylation is a critical regulatory switch for modulation of cardiac contraction^44,45^. Previously, TnT was identified as an A-kinase anchoring protein (AKAP) which brings PKA at the thin filaments into close proximity with its sarcomeric substrates, including TnI^30^. Generally, beta-adrenergic stimulation leads to a cell- wide increase of cAMP, which is important for local regulation of cAMP-PKA responses^14^. Using a sarcomeric TnI-localized FRET biosensor, we determined dysregulation of local sarcomeric cAMP/PKA pools in presence of the TnT-R173W mutation. DCM iPSC-CMs attempt an upregulation of local sarcomeric cAMP, likely to compensate for reduced PKA binding in presence of the TnT-R173W mutation, which limits TnI phosphorylation at local sarcomere microdomains, thereby diminishing contractility. On the other hand, we found a substantial upregulation of sarcomeric PDE activity in DCM TnT-R173W iPSC-CMs, in line with a recent report proposing upregulation of PDE isoform expression as a basis for deficient beta-adrenergic activation in DCM iPSC-CMs^10^. Thus, the observed slight increase in sarcomeric cAMP levels in DCM TnT-R173W iPSC-CMs may present a compensatory reaction and long- term-adaptation of cardiomyocyte signaling. Importantly, sarcomeric cAMP alterations could not counterbalance reduced PKA binding to TnT in presence of TnT-R173W, which may contribute to the impaired contractility observed in DCM TnT-R173W iPSC-CMs.

Together, these findings indicate a highly sensitive balance of sarcomere microdomain regulation, which is disrupted in presence of DCM TnT-R173W. Consequently, as our results show, sarcomere-cytoskeleton filament attachment is mediated via MYH7 and AMPK and is impaired in DCM iPSC-CMs. Binding of cytoskeleton attachment proteins, such as MYH7 and filamin-C, with TnT was significantly diminished in presence of the TnT-R173W mutation. Also, sarcomere attachment with plasma membrane junctions appears to be impaired in DCM iPSC-CMs compared to healthy controls. Particularly, TnT binding to MYH7 implies a critical link of sarcomere protein alignment and organization to cytoskeleton filament stability and function. Our data suggest that disturbed binding of TnT-R173W to MYH7 directly destabilizes MYH7-AMPK interactions and AMPK-mediated modulation of cytoskeleton integrity. AMPK, a protein which assists cytoskeleton remodeling, was found to regulate sarcomere-cytoskeleton filament interactions in iPSC-CMs. Small molecule-based AMPK activation could ameliorate disruption of sarcomere-cytoskeleton protein interaction sites in DCM TnT-R173W iPSC-CMs. Importantly, activation of AMPK leads to improved contractility and sarcomeric protein alignment in DCM iPSC-CMs. Our findings suggest that in DCM TnT-R173W iPSC-CMs, disturbed interactions of TnT and resulting reduced sarcomere protein alignment and interactions require a substantial activation of AMPK to overcome the consequences of the TnT-R173W mutation. In line with a previous publication^46^, we observe that AMPK activation improves contractility in cardiomyocytes. In our study, we utilize a recently developed, direct AMPK activator, A-769662, which is not expected to exert side-effects on e.g. metabolism-related enzymes^35,47,48^.

Figuratively, these signaling events resemble a “molecular seesaw”, in which the DCM mutation causes disturbed binding of TnT-R173W to MYH7, which in turn directly translates into impaired MYH7-AMPK interactions and AMPK-mediated modulation of cytoskeleton integrity. Conversely, activation of AMPK causes a recovery shift in the opposite “seesaw” direction, by restoring cytoskeleton-sarcomere interactions and thus partially recovering sarcomere protein alignment as well as contractility. Overall, our study reveals impaired sarcomere microdomain regulation in DCM iPSC-CMs such as disrupted binding of mutated TnT-R173W with tropomyosin as well as PKA, affecting sarcomeric protein alignment and local sarcomeric cAMP/PKA regulation. These molecular functions are modulated by AMPK and contribute to reduced sarcomere protein regularity and defective force generation observed in DCM iPSC-CMs. Moreover, our findings provide novel insight into the tightly regulated interplay of sarcomeric microdomains with cytoskeleton- as well as plasma membrane junctions in iPSC-CMs from DCM patients. Further studies of these molecular functions may assist the development of novel therapeutic strategies exploiting these regulatory mechanisms to combat cardiac disease.

## Materials and Methods

### Cardiac differentiation of iPSCs

Human iPSCs were grown to 80–90% confluence using matrigel-coating and chemically defined E8 medium^26,49^. Subsequently, iPSCs were differentiated into beating cardiomyocytes with a small molecule–based monolayer method described previously^24,25^. In short, a GSK inhibitor, Chir (Selleckchem) was applied for 48 h, followed by addition of the canonical Wnt-signaling inhibitor, IWR2 (Selleckchem). Beating iPSC-derived cardiomyocytes (iPSC-CMs) were observed from day 7–10 onwards. Following differentiation, human iPSC-CMs were cultured in RPMI medium plus B-27 Supplement (Life Technologies). Human iPSC-CMs expressed typical cardiac markers such as cardiac troponin T (TNNT2), sarcomeric a-actinin (SAA), and myosin light chain 2a (MYL2a). Following 25 days of cardiac differentiation, beating iPSC-CM monolayers were dissociated using Accutase and plated in the required assay format. All protocols required for this study were approved by the Goettingen University Ethical Board. Informed consent was obtained from all participants and all research was performed in accordance with relevant guidelines and regulations.

### CRISPR/Cas9 genetic engineering and plasmid generation

CRISPR/Cas9-mediated gene editing was performed as described before^50–53^. Briefly, ATG knock-out of troponin T was performed by oligonucleotide annealing the sgRNA sequences (5′- GACCATGTCTGACATAGAAG-3′, 5′-CTTCTATGTCAGACATGGTC-3′), subcloning into the pSpCas9(BB)-2A-Puro plasmid and subsequent iPSC transfection and single clone isolation. pcDNA 3.1 plasmids carrying the full-length TNNT2-C-terminal-DYK sequence were purchased from GenScript. The TNNT2-R173W was generated by PCR-based site-directed mutagenesis as described before^54^.

### Immunoprecipitation and immunoblotting

TnT-specific antibody (Thermo Fisher Scientific) was immobilized on IgG1 Protein G Sepharose beads (GE Healthcare) according to the manufacturer’s instructions. Subsequently, iPSC-CMs lysates were prepared in immunoprecipitation binding and wash buffer as described before^54^ with protease and phosphatase inhibitors. Eluted protein solutions as well as lysate input were subjected to immunoblot. For visualization of phosphorylated TnI (p-TnI), a phospho-TnI- Ser23/24 antibody (Cell Signaling cat. no. 4004 S) was used. In addition, phos-tag acrylamide was used^55,56^. Non-phosphorylated and phosphorylated cTnI species were separated in one- dimensional SDS-PAGE with containing phos-tag acrylamide and transferred to Western blots.

### Generation of engineered heart muscle (EHM) tissues

Human ESC-CMs (2.5 × 10^6^) were first gently mixed on ice with collagen type I and serum-free EHM medium and casted into custom- made molds according to a previously published protocol^57^. Following 5 days condensation in casting molds, EHMs were transferred onto mechanical stretchers for functional maturation for an additional 12–14 days. EHM media was changed every other day. Contractile forces generated by EHMs were measured in organ baths in Tyrode’s solution containing 1.8 mmol/L calcium under 1.5 Hz field stimulation.

### Statistical analysis

Statistical significance was determined using an unpaired Student’s t-test for comparison of two normally distributed data sets. A one-way or two-way ANOVA was used as appropriate to compare multiple data sets, together with post-hoc tests for pairwise comparisons according to the Sidak or Tukey methods, depending on the properties of the data sets. For non- parametric tests, Mann-Whitney- or Kruskal-Wallis tests were used together with Dunn’s post-hoc multiple-comparison test. P-values < 0.05 were considered as statistically significant. Data are presented as mean ± standard error of mean (sem).

An expanded Methods section is available in the online Data Supplement.

## Supplementary information

Supplementary information

Supplementary movie 1

Supplementary movie 2

Supplementary movie 3
