# Supplementary material for: Troponin destabilization impairs sarcomere-cytoskeleton interactions in iPSC-derived cardiomyocytes from dilated cardiomyopathy patients
Source: Sci Rep. 2020 Jan 14;10:209. doi: 10.1038/s41598-019-56597-3 (PMC6959358; doi:10.1038/s41598-019-56597-3)
Supplement: Supplementary file 1 — Supplementary information [file 41598_2019_56597_MOESM1_ESM.pdf]

SUPPLEMENTAL MATERIALS

Troponin destabilization impairs sarcomere-cytoskeleton interactions in iPSC-derived cardiomyocytes from dilated cardiomyopathy patients

Yuanyuan Dai[1,3], Asset Amenov[1,3], Nadezda Ignatyeva[1,3], Andreas Koschinski[5], Hang Xu[1,3], Poh Loong Soong[2,3], Malte Tiburcy[2,3], Wolfgang A. Linke[1,3, 4], Manuela Zaccolo[5], Gerd Hasenfuss[1,3], Wolfram-Hubertus Zimmermann[2,3], Antje Ebert[1,3]

[1]Heart Center, Department of Cardiology and Pneumology; [2]Institute of Pharmacology, University Medical Center, University of Goettingen, Robert-Koch-Str. 40, 37075 Goettingen, Germany; [3]DZHK (German Center for Cardiovascular Research), partner site Goettingen, Germany; [4]Institute of Physiology II, University of Muenster, Germany; [5]Department of Physiology, Anatomy and Genetics, University of Oxford, Oxford OX1 3PT, UK.

SUPPLEMENTAL METHODS

Culture and maintenance of human iPSCs. The human iPSC lines described earlier (*1*-*3*) were grown on Matrigel-coated plates (ES qualified, BD Biosciences, San Diego) as described previously (*4, 5*) using chemically defined E8 medium (*4*). Culture medium for iPSCs was changed daily. Cells were passaged every 4 days using Accutase (Global Cell Solutions). Healthy control and DCM iPSCs as published in (*1, 2*) were a kind gift from Joseph C. Wu (Stanford University, CA). Protocols for human biomaterial studies required for this study were approved by the Goettingen University Ethical Board.

Pluripotency marker analysis. Human iPSC colonies were grown in 6-well plate format (Sigma Aldrich) and stained as described previously (*1, 5*) for Oct3/4 (Cell Signaling Technology), Tra-1–60 (Sigma-Aldrich/Chemicon), SSEA4 (Santa Cruz Biotechnology) and Nanog (Santa Cruz Biotechnology). Images were acquired with a brightfield microscope (Leica).

Flow cytometry. Assessment of TnT expression in human iPSC-CMs by flow cytometry was performed as described before (*6*). Briefly, iPSC-CMs were detached as described above, and subsequently fixed and permeabilized (*5, 6*). Cells were incubated with a primary anti-cardiac TnT antibody (Thermo Scientific) for 2 h at 4°C, and a secondary Alexa Fluor-488 antibody (Life Technologies) for 45 min at 4°C. Cells were washed and analyzed by fluorescent activated cell sorting (FACS; BD Aria II), and data were analyzed using FlowJo software.

Immunofluorescence staining and confocal microscopy. Differentiated iPSC-CMs were passaged on matrigel-coated glass coverslips. Cells were stained as described before with mouse

anti-human antibodies for cardiac troponin T (Thermo Scientific and Abcam) and sarcomeric alpha-actinin (Sigma). DAPI was used for staining of nuclei. Coverslips were mounted on glass slides using Fluoromount-G. Pictures were taken with 10x, 40x (plan apochromat), and 63x (plan apochromat oil) objectives using an inverted confocal microscope (Carl Zeiss, LSM 710 Meta, Göttingen, Germany) and ZEN software (Carl Zeiss).

Quantification of protein co-localization. Confocal images generated as described above were analyzed for protein co-localization analysis using the ImageJ function Coloc2. Background correction was performed using Fiji-ImageJ. For individual cells, ROIs were selected. Coloc2 was used for background subtraction, and analysis for obtaining Manders´ correlation was performed as described before (*7-9*).

Co-immunoprecipitation with DYK-tagged TnT and immunoblot. HEK 293T cells overexpressing in a pcDNA 3.1 backbone either TnT-WT-DYK, TnT-R173W-DYK or DYK tag alone as a negative control were lysed and input was kept for analysis. Cell lysates were bound with DYK-antibody-decorated magnetic beads and subsequently, immobilized TnT-DYK was incubated for co-immunoprecipitation (*10*) with cell lysate from healthy control iPSC-CMs. The bound fraction was analyzed by immunoblot. The following antibodies were purchased from Abcam: Tropomyosin (ab7785), troponin C (ab137130), troponin I (ab52862), phospholamban (ab2865). Myosin heavy chain antibody was purchased from DSHB.

PDE activity assay. Human iPSC-CM lysates were prepared as described (*5*). Sarcomere-containing fractions were obtained by pelleting pre-cleared lysates (*10*). The cytosolic supernatant

was transferred to a new tube and analyzed separately. Both cytosolic and sarcomere-containing fractions were subsequently analyzed using a PDE activity assay kit (Abcam, ab139460) according to the manufacturer´s instructions.

Analysis of AMPK activity via phosphorylation of T172. Human iPSC-CM lysates were prepared as described (*5*) from healthy control (n=2 cell lines) and DCM (n=3 cell lines) groups. To determine AMPK activity based on phosphorylation of Thr-172 in the AMPK catalytic subunit, cell lysates were incubated with A-769662 or control vehicle (DMSO) and subsequently subjected to immunoblot analysis.

Fluorescent resonance energy transfer (FRET) measurements and analysis. All FRET-measurements were performed with a Nikon Eclipse FN-1 microscope. An Opto-Led fluorescent light source (Cairn-Research) was used for excitation at 436 ± 25 nm, the excitation / emission dichroic was 455 nm (long pass). Emitted light was split with a Dual-View beam splitter (Optical Insights) and recorded with a CoolSnap HQ^2 camera (Photometrix). Emission light was split with a 505 nm longpass dichroic and was then filtered at 480 ± 15 nm for CFP emission and 535 ± 20 nm for YFP emission. Extracellular buffer for the measurements was a modified and CO_2 supplemented Ringer-solution with NaCl (140 mM), KCl (3 mM), $MgCl_2$ (2 mM), $CaCl_2$ (2 mM), Glucose (15 mM), HEPES (10 mM), pre-adjusted to pH 7.2 with NaOH, then supplemented with $NaHCO_3$ (10 mM) and finally pH-adjusted to pH 7.2. Acquisition and analysis were performed using Optofluor software (Cairn Research). All ratios were calculated as emission at 535 nm / emission at 480 nm. The TPNI-CUTie FRET-sensor was developed before (*11*). Adenoviruses were used to infect cells 24-48h before the measurements.

SUPPLEMENTAL FIGURE LEGENDS

Supplementary Figure 1: Characterization of DCM and TnT-KO iPSC-CMs. (A) Sanger sequencing chromatogram of genomic DNA from healthy control and DCM-TnT-R173W iPSCs. Arrow indicates site of mutation. (**B**) Strategy for generation of a troponin T knock-out in iPSC via CRISPR/Cas9-mediated targeting of *TNNT2* exon 2. Underlined corresponds to sequence of the sgRNA employed for targeting of exon 2 (red highlight, ATG). (**C**) Immunohistochemistry and wide-field imaging show expression of the indicated pluripotency markers. (**D**) Healthy control and DCM iPSC-CMs express standard sarcomeric marker proteins. Expression of MYL2 (MYL) and MYH7 is reduced in TnT-KO iPSC-CMs. *$P<0.05$, **$P<0.01$, ***$P<0.001$ and ns, not significant, as calculated by Kruskal-Wallis test and Dunn's multiple comparisons test. (**E**) Representative analysis of TnT expression in human iPSC-CMs via flow cytometry. (**F**) Immunohistochemistry for sarcomeric alpha-actinin and cardiac troponin T, followed by confocal imaging was performed (shown in Fig 1A) and sarcomeric alpha-actinin and TnT signals were analyzed by Pearson´s correlation and Fast Fourier transformation, to obtain correlation coefficients in DCM iPSC-CMs (94 cells) than in healthy controls (n=78 cells). The difference between the groups is not statistically significant as calculated by Mann-Whitney test. TnT KO iPSC-CMs could not be reliably analyzed due to lack of signal for TnT. Data are expressed as mean ± sem.

Supplementary Figure 2: Expression levels of TNNT2, TNNC1, TNNI3 and TPM1 are not significantly altered in TnT-R173W iPSC-CMs. (A-D) Expression of TNNT2 (**A**), TNNC1(**B**), TPM1 (**C**) and TNNI3 (**D**) was determined at the mRNA level via quantitative real-time PCR (qRTPCR). WT control vs DCM, not significant (ns) and ***$P<0.001$ towards all other groups, as

calculated by Kruskal-Wallis test and Dunn's multiple comparisons test. (**E-H**) Expression levels of the troponin complex were comparable in DCM-R173W iPSC-CMs and healthy controls. Immunoblot analysis for protein expression of troponin T (TnT) (**E**), troponin C (TnC) (**F**), tropomyosin (Tm) (**G**), and troponin I (TnI) (**H**) was performed for DCM R173W iPSC-CMs compared to healthy control- (WT) and TnT KO iPSC-CMs. Control (WT), n=3 cell lines; DCM (TnT-R173W), n=3 cell lines; TnT KO, n=1 cell line. Bargraphs display averages of n=3 experiments and shown below are representative immunoblots; ns, not significant as calculated by one-way ANOVA and multiple comparisons tests (Dunn and Sidak methods). Data are expressed as mean ± sem.

Supplementary Figure 3, relating to Figure 2: Interactions within the troponin complex are affected by the DCM mutation TnT-R173W. (A-C) Protein-protein interactions within the troponin-tropomyosin complex were assessed by immunoprecipitation using a TnT-specific antibody. (**A**) Equal amounts of TnT-WT and TnT-R173W were immunoprecipitated from lysates of healthy control and DCM. No substantial binding is detected for TnT-KO iPSC-CMs (negative control). TnC (**B**), Tm (**C**) and TnI (**D**) binding to TnT is shown. Tm-binding to TnT-R173W is reduced in DCM TnT-R173W iPSC-CMs, compared to WT controls. TnT KO iPSC-CMs are employed as negative control. Bargraphs are shown for control (WT, n= 3 cell lines), DCM (TnT-R173W, n=3 cell lines), and TnT KO (n=1 cell line). TnT, averages of n=8 experiments; TnC, averages of n=8 experiments; Tm, averages of n=7 experiments; and TnI, averages of n=2 experiments. Representative immunoblots are shown. ns=not significant as calculated by one-way ANOVA and Dunn´s multiple comparison test. Data are expressed as mean ± sem.

Supplementary Figure 4, relating to Figure 2-3: Disturbed interactions within the troponin complex in presence of DCM-TnT-R173W. (**A**) Input for TnT-WT-DYK and TnT-R173W-DYK (MUT) as well as DYK-negative control (NC) from HEK cell lysates prior to coupling to DYK-decorated beads; and input for human iPSC-CM WT cell lysate used for co-immunoprecipitation of TnC, Tm, TnI as well as PKA. GAPDH was used as a loading control. (**B**) Phos-tag gel analysis of DCM- and healthy control iPSC-CM cell lysates is shown. TnI phosphorylation in DCM TnT-R173W is reduced compared to healthy control iPSC-CMs. Shown is 2-P-phosphorylated TnI as reported previously (*50*). Averages of 3 independent experiments are shown for control (n=3 cell lines), DCM (n=3 cell lines); ***P<0.001 (Mann-Whitney test). Data are shown as mean ± sem. Representative membrane scans are shown. Reduced PKA-mediated TnI phosphorylation was also analyzed with a TnI-Ser 23/24 specific antibody (Figure 3A). (**C-D**) FRET-based analysis of cAMP levels in TnT-KO iPSC-CMs versus WT controls. (**C**) Disorganized sarcomeric structure in TnT-KO iPSC-CMs results in corresponding mislocalization of the sarcomeric FRET-sensor TPNI-CUTie. Respectively, no significant difference in sarcomeric cAMP levels is detected in TnT-KO iPSC-CMs, compared to WT. Representative images are shown; scale bar, 10 µm. (**D**) Quantification of TPNI-CUTie FRET analysis shown in (**C**). No significant difference is detected between WT control and TnT-KO (P =0.0981). WT iPSC-CMs (healthy control), n=45 cells; TnT-KO iPSC-CMs, n=89 cells. ns = not significant (Student´s t-test). Data are shown as mean ± sem.

Supplementary Figure 5: Total cytosolic PDE activity is elevated in DCM TnT-R173W iPSC-CMs. (**A**) Adenylyl cyclase protein expression levels are not significantly changed in DCM iPSC-CMs and healthy controls as well as TnT-KO iPSC-CMs. Bargraphs indicate averages of n=2

experiments for n=2 cell lines (WT, DCM) and n=1 cell line (TnT-KO), representative immunoblots are shown below. Differences are not statistically significant (one-way ANOVA and Dunn´s multiple comparison test). (**B-C**) Measurements of cytosolic PDE activity in DCM iPSC-CMs compared to healthy controls as well as TnT-KO iPSC-CMs, using a PDE activity assay kit (Abcam). (**B**) Measurement of PDE activity via 5´AMP release, using a standard curve for absorbance at 620 nm. (**C**) Differences in total cytosolic PDE activity were not significantly altered between DCM- and TnT-KO iPSC-CMs, compared to healthy controls. Averages of n=2 experiments for n=2 cell lines (WT, DCM) and n=1 cell line (TnT-KO) are shown. Differences are not statistically significant, as calculated by Kruskal-Wallis test and Dunn's multiple comparisons test. Data are shown as mean ± sem.

Supplementary Figure 6, relating to Figure 4: Interaction of TnT with cytoskeleton filament proteins. Input for TnT IP from healthy control (WT), DCM (TnT-R173W) and TnT-KO iPSC-CMs is shown. Input was collected from cell lysates prior to coupling to TnT antibody-decorated beads (**A**) mouse TnT, relating to Figure 4C-E and (**B**) Immunoblot analysis for filamin-C expression in DCM-TnT-R173W iPSC-CMs and WT controls using input fractions from immunoprecipitation of TnT with filamin-C shown in Fig 4C-E. A representative membrane scan is shown. (**C**) Quantification of (**B**), n=3 experiments. *P*= not significant (one-way ANOVA and Dunn's multiple comparisons test). Data are shown as mean ± sem.

Supplementary Figure 7, relating to Fig. 5: Interaction of TnT with cytoskeleton filament proteins. (**A**) Input for relevant proteins in TnT IP with MYH7 is shown for healthy control (WT), DCM (TnT-R173W) and TnT-KO iPSC-CM cell lysates. Input was collected from cell lysates

prior to coupling to TnT antibody-decorated beads (mouse TnT). GAPDH was used as a loading control. (**B**) Western blot indicates total protein expression levels of AMPK to be comparable in DCM and healthy control iPSC-CMs. Quantification of 2 experiments is shown. Control, n=2 cell lines; DCM, n=3 cell lines. *P* = not significant (Mann-Whitney test); data are shown as mean ± sem.

Supplementary Figure 8, relating to Fig. 6: AMPK inhibition in WT iPSC-CMs results in impaired contractility as observed in DCM iPSC-CMs. Motion-traction analysis of WT iPSC-CMs treated for 24h with an AMPK inhibitor, BML-275. (**A**) Time-to-peak; (**B**) Contraction amplitude; (**C**) Contraction duration. Quantification is shown for n=2 cell lines; ***P*<0.01 and ****P*<0.001 as calculated by Student´s t-test. Data are shown as mean ± sem.

Supplementary Table 1, relating to Fig. 1: Numerical values represented in the error bars in Fig. 1I (standard error of mean) showing force-of-contraction measurements in EHMs from WT control and DCM TnT-R173W iPSC-CMs. n=8 EHMs per group. *P < 0.05 and ns, not significant as calculated by two-way ANOVA and Tukey's post-hoc test.

REFERENCES

1. N. Sun *et al.*, Patient-specific induced pluripotent stem cells as a model for familial dilated cardiomyopathy. *Sci Transl Med* **4**, 130ra147 (2012).
2. F. Lan *et al.*, Abnormal calcium handling properties underlie familial hypertrophic cardiomyopathy pathology in patient-specific induced pluripotent stem cells. *Cell Stem Cell* **12**, 101-113 (2013).

3. H. Wu *et al.*, Epigenetic Regulation of Phosphodiesterases 2A and 3A Underlies Compromised beta-Adrenergic Signaling in an iPSC Model of Dilated Cardiomyopathy. *Cell Stem Cell* **17**, 89-100 (2015).
4. G. Chen *et al.*, Chemically defined conditions for human iPSC derivation and culture. *Nat Methods* **8**, 424-429 (2011).
5. A. D. Ebert *et al.*, Characterization of the molecular mechanisms underlying increased ischemic damage in the aldehyde dehydrogenase 2 genetic polymorphism using a human induced pluripotent stem cell model system. *Sci Transl Med* **6**, 255ra130 (2014).
6. P. E. de Almeida *et al.*, Transplanted terminally differentiated induced pluripotent stem cells are accepted by immune mechanisms similar to self-tolerance. *Nat Commun* **5**, 3903 (2014).
7. E. M. M. Manders, V. F.J., A. J.A., Measurement of co-localization of objects in dual-colour confocal images *Journal of Microscopy* **169**, 375-382 (1993).
8. J. Oswald *et al.*, Leptin affects filopodia and cofilin in NK-92 cells in a dose- and time-dependent manner. *Eur J Histochem* **62**, 2848 (2018).
9. V. Zinchuk, O. Zinchuk, T. Okada, Quantitative colocalization analysis of multicolor confocal immunofluorescence microscopy images: pushing pixels to explore biological phenomena. *Acta Histochem Cytochem* **40**, 101-111 (2007).
10. A. D. Ebert *et al.*, Tec-kinase-mediated phosphorylation of fibroblast growth factor 2 is essential for unconventional secretion. *Traffic* **11**, 813-826 (2010).
11. N. C. Surdo *et al.*, FRET biosensor uncovers cAMP nano-domains at beta-adrenergic targets that dictate precise tuning of cardiac contractility. *Nat Commun* **8**, 15031 (2017).

SUPPLEMENTAL FIGURES

Troponin destabilization impairs sarcomere-cytoskeleton interactions in iPSC-derived cardiomyocytes from dilated cardiomyopathy patients

Yuanyuan Dai[1,3], Asset Amenov[1,3], Nadezda Ignatyeva[1,3], Andreas Koschinski[5], Hang Xu[1,3], Poh Loong Soong[2,3], Malte Tiburcy[2,3], Wolfgang A. Linke[1,3, 4], Manuela Zaccolo[5], Gerd Hasenfuss[1,3], Wolfram-Hubertus Zimmermann[2,3], Antje Ebert[1,3]

[1]Heart Center, Department of Cardiology and Pneumology; [2]Institute of Pharmacology, University of Goettingen, Robert-Koch-Str. 40, 37075 Goettingen, Germany; [3]DZHK (German Center for Cardiovascular Research), partner site Goettingen, Germany; [4]Institute of Physiology II, University of Muenster, Germany; [5]Department of Physiology, Anatomy and Genetics, University of Oxford, Oxford OX1 3PT, UK.

Supplementary Figure 1

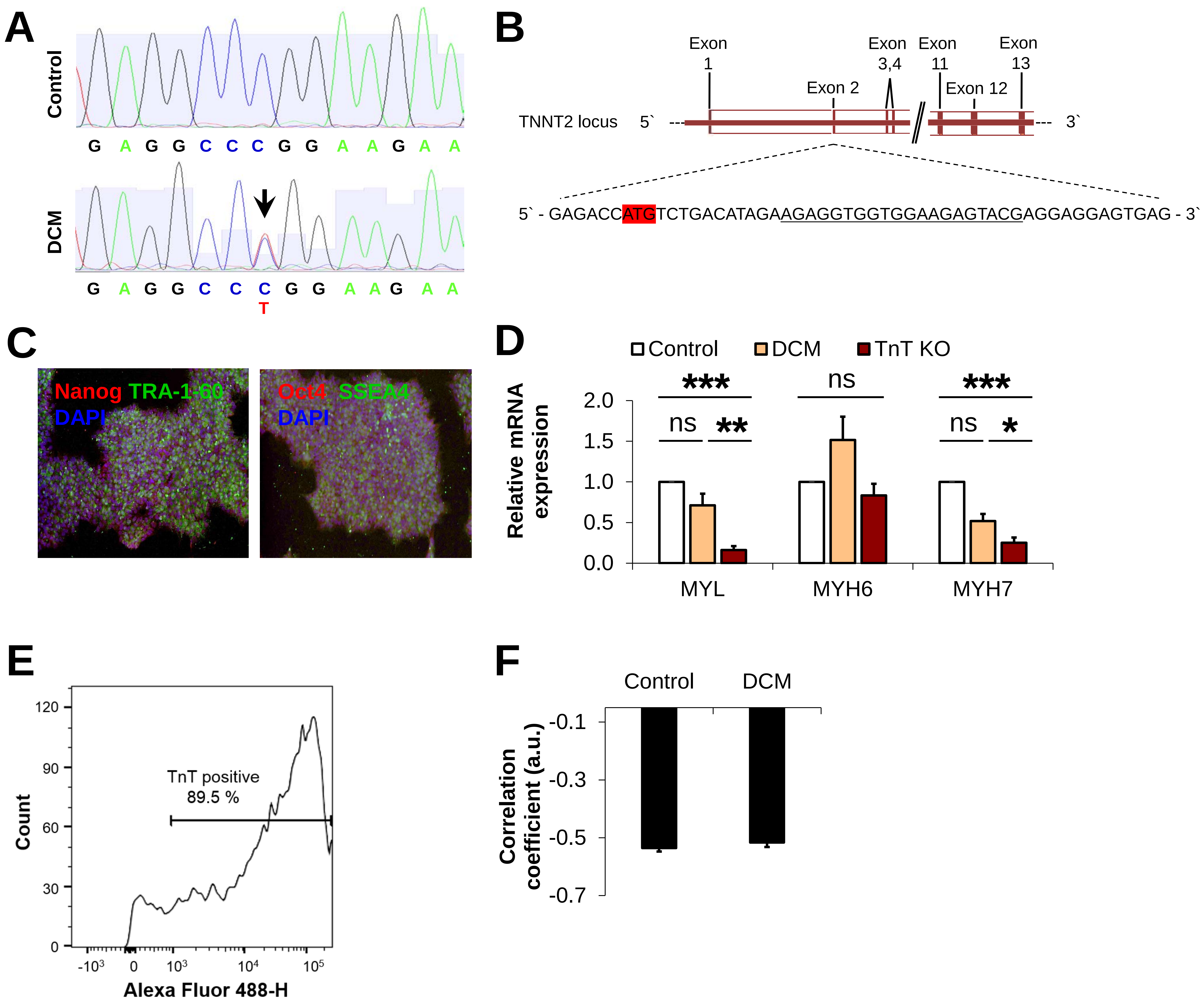

Supplementary Figure 2

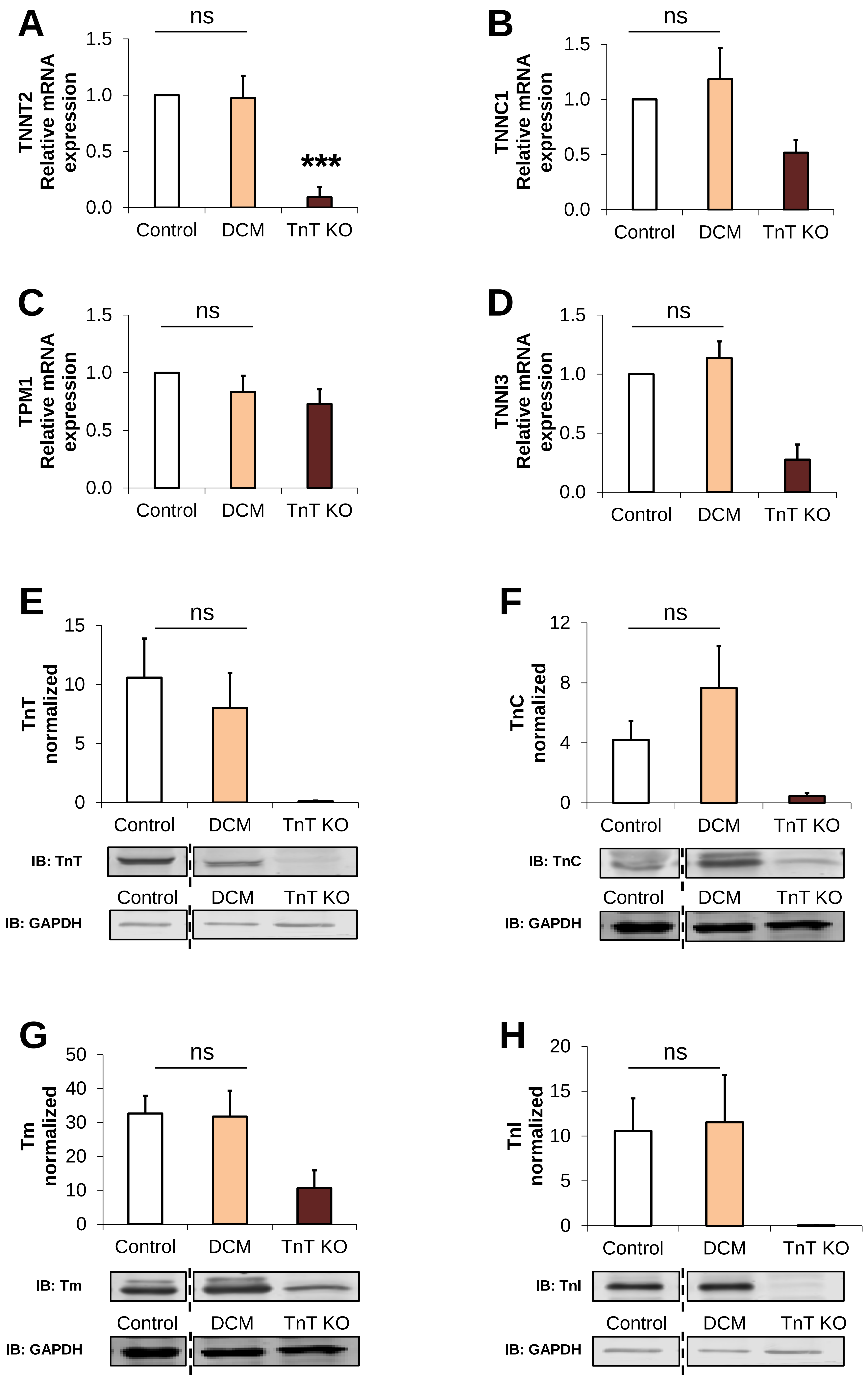

Supplementary Figure 3

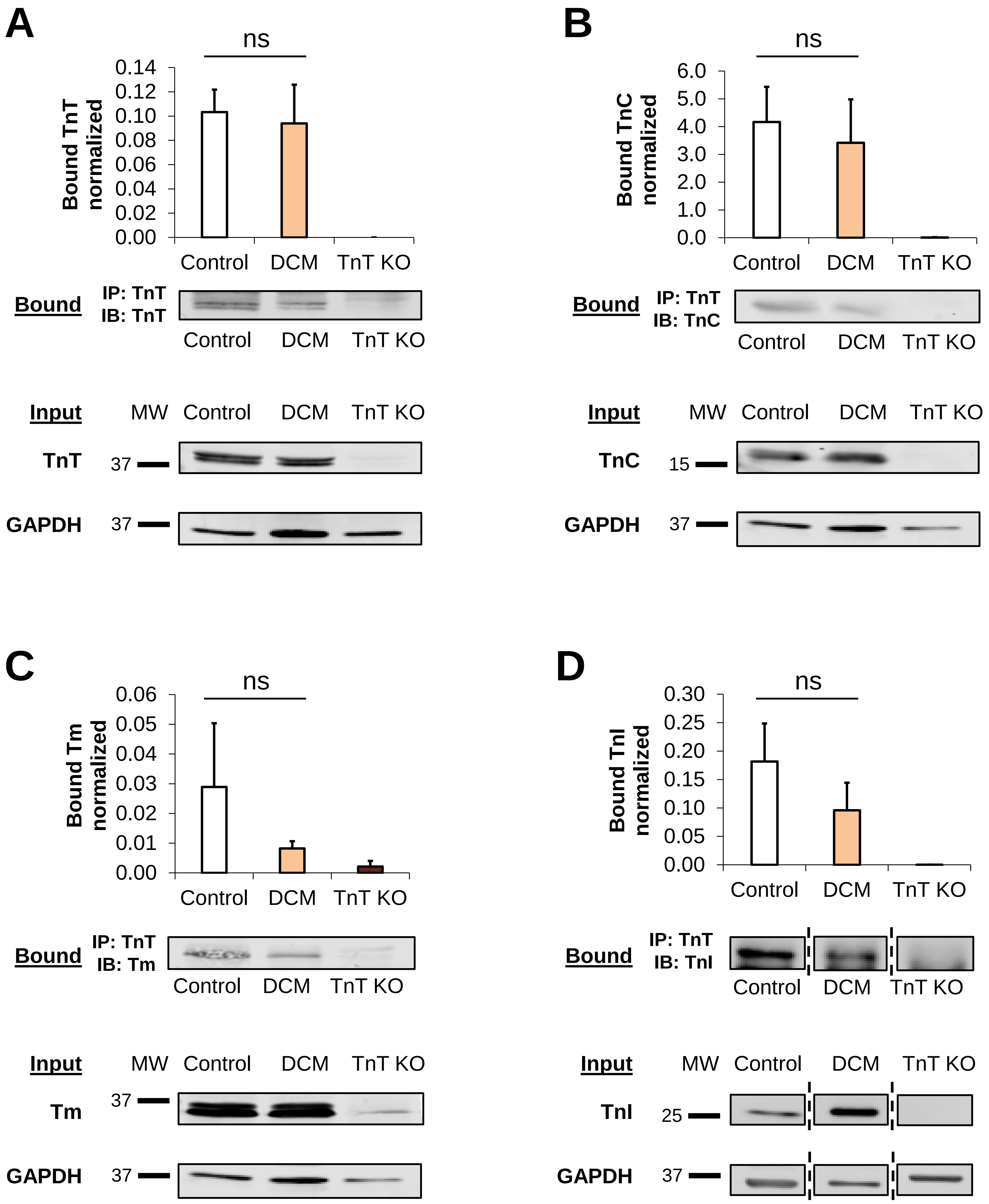

Supplementary Figure 4

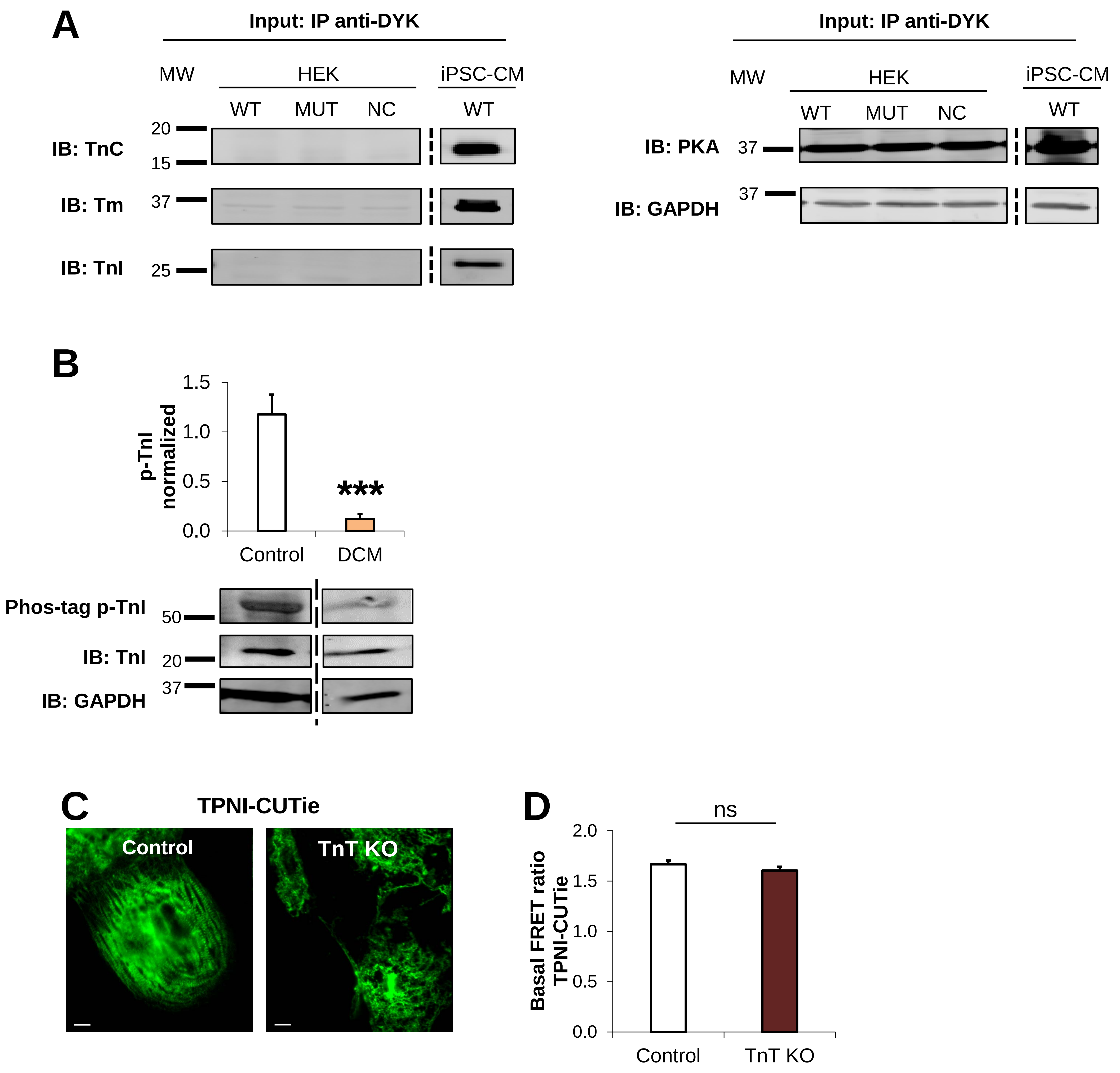

Supplementary Figure 5

A

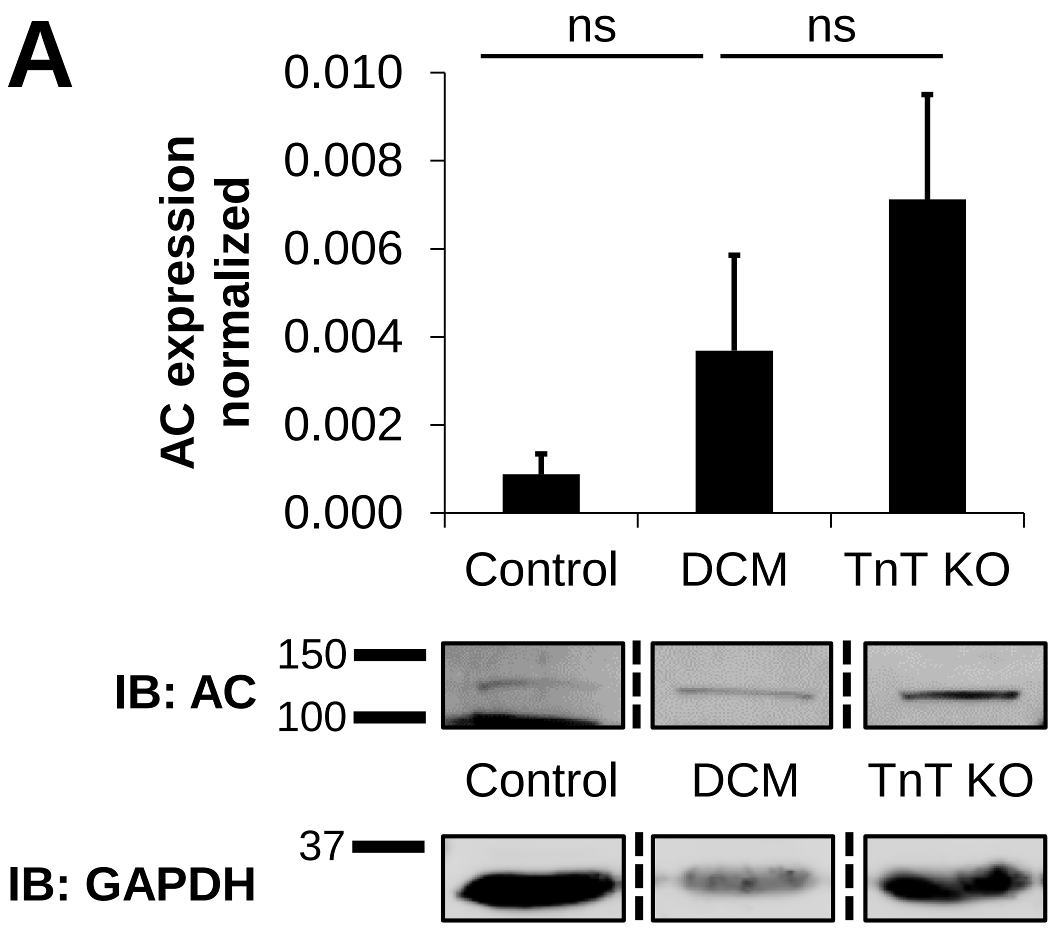

B

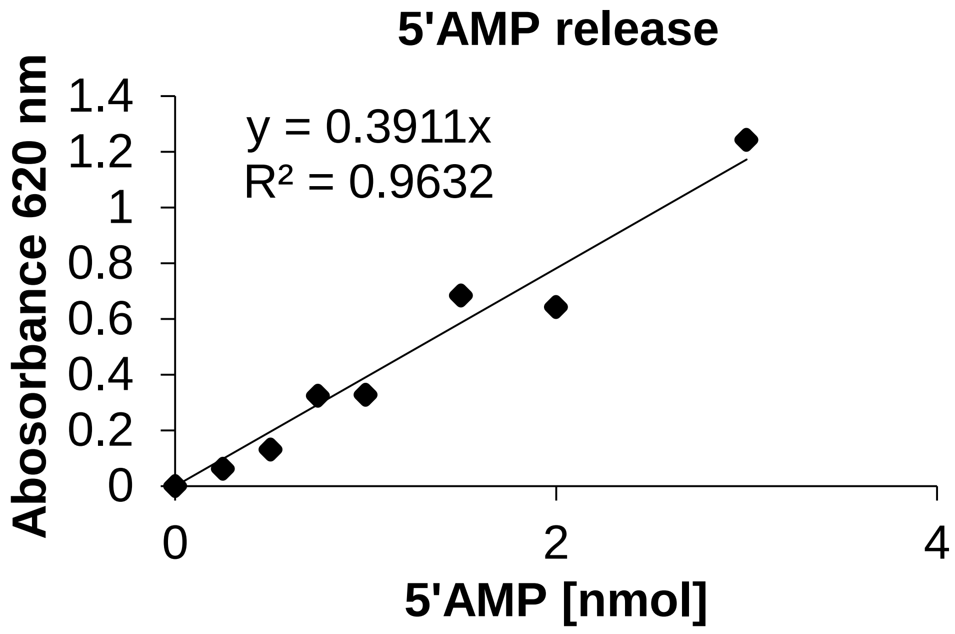

C

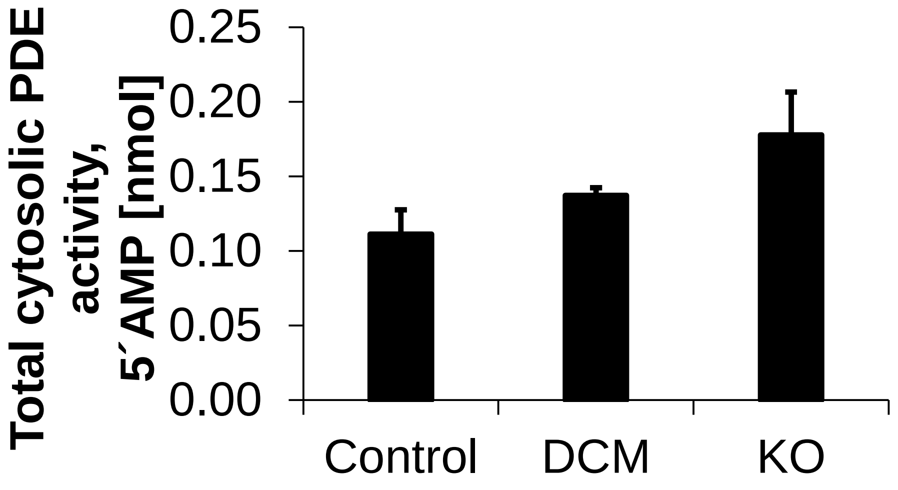

Supplementary Figure 6

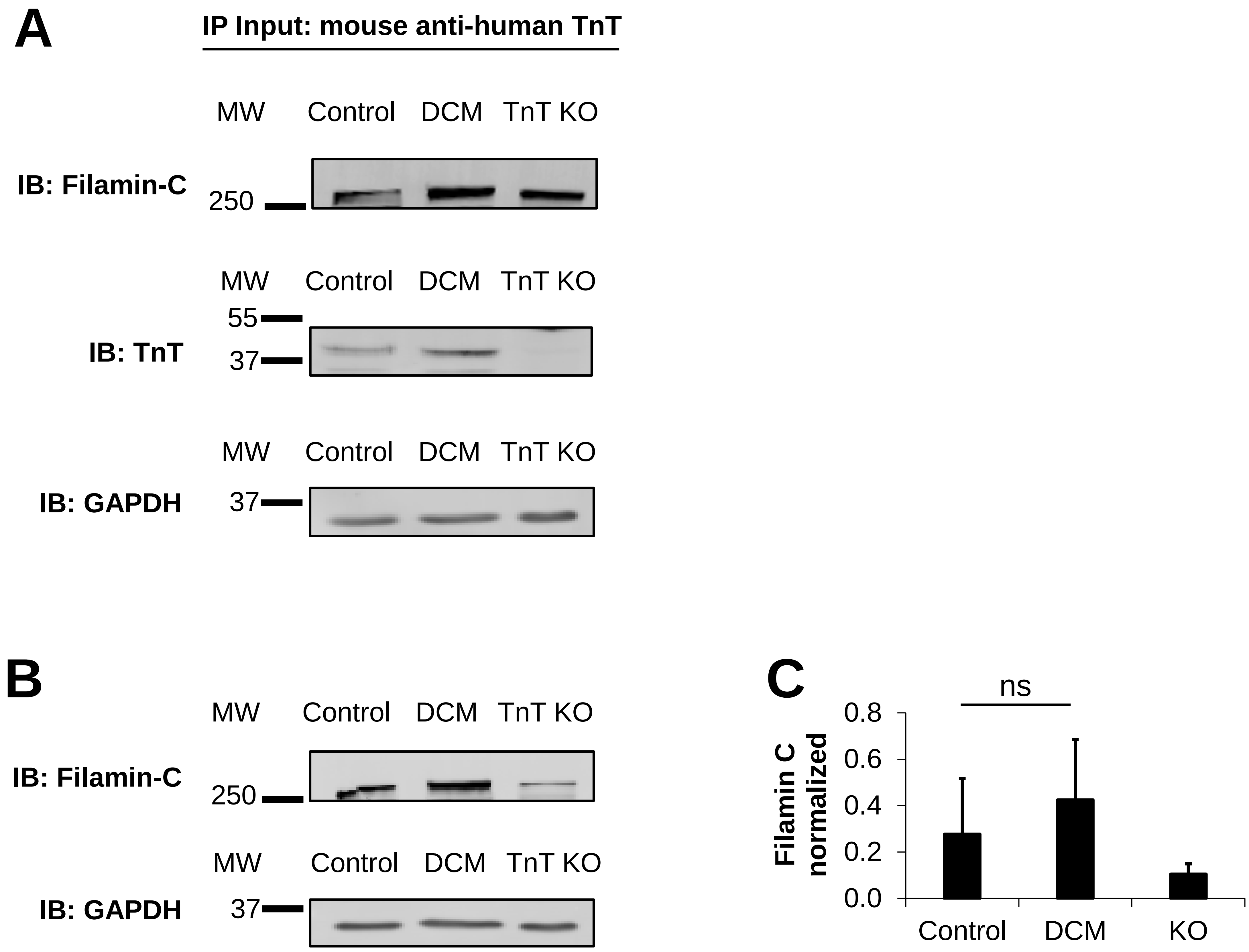

Supplementary Figure 7

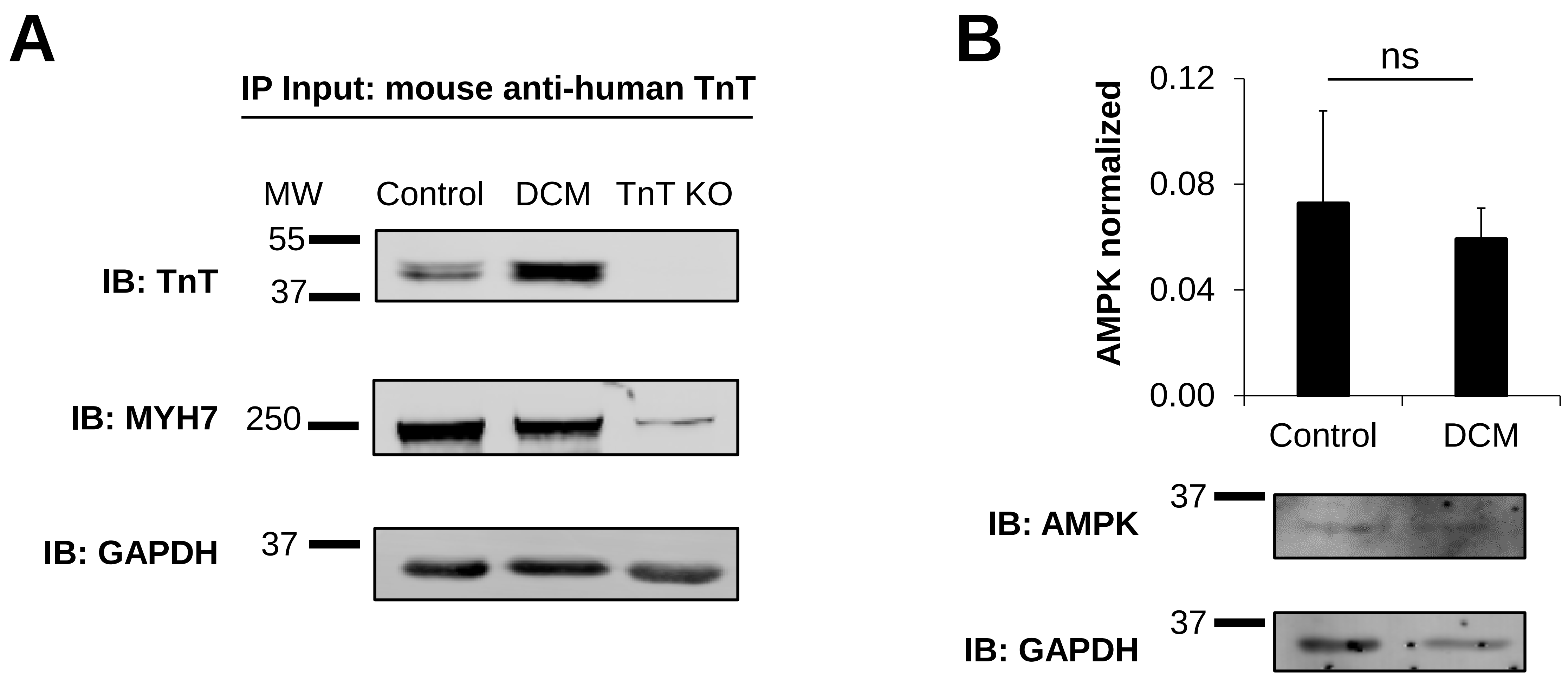

Supplementary Figure 8

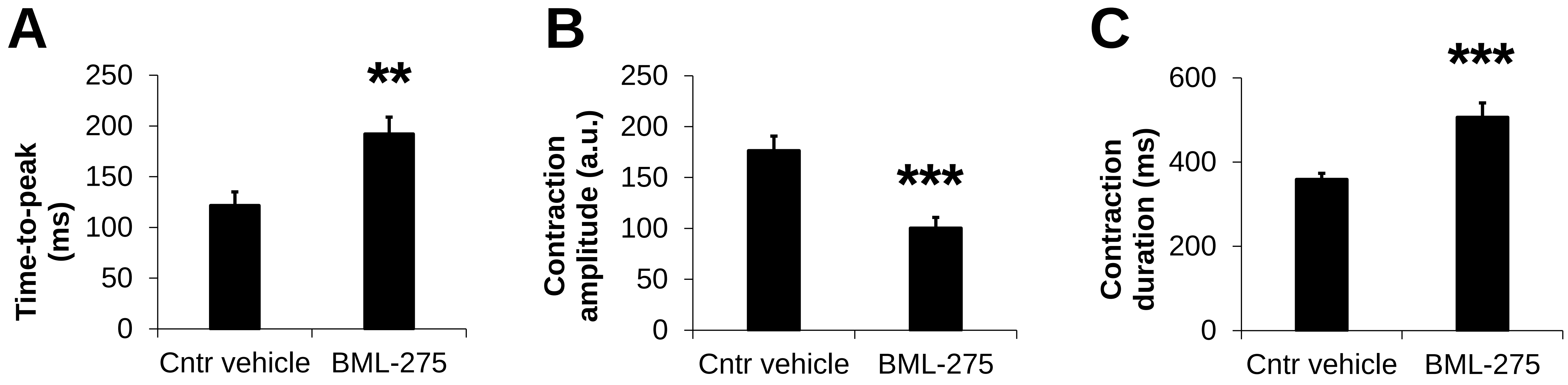

Supplementary Table 1

Ca^{2+}[mmol/l]	Control (WT) EHMs			DCM (TnT-R173W) EHMs		
	Mean	SEM	N	Mean	SEM	N
0.2	0.079	0.014	8	0.025	0.003	8
0.4	0.194	0.036	8	0.066	0.006	8
0.8	0.458	0.077	8	0.181	0.023	8
1.2	0.660	0.096	8	0.267	0.029	8
1.6	0.817	0.081	8	0.327	0.023	8
2	0.933	0.096	8	0.365	0.021	8
2.4	0.984	0.096	8	0.389	0.019	8
2.8	1.020	0.100	8	0.404	0.019	8
3.2	1.052	0.102	8	0.405	0.019	8
4	1.102	0.102	8	0.407	0.021	8
